# Halophytic Clonal Plant Species: Important Functional Aspects for Existence in Heterogeneous Saline Habitats

**DOI:** 10.3390/plants12081728

**Published:** 2023-04-21

**Authors:** Gederts Ievinsh

**Affiliations:** Department of Plant Physiology, Faculty of Biology, University of Latvia, 1 Jelgavas Str., LV-1004 Rīga, Latvia; gederts.ievins@lu.lv

**Keywords:** clonal plants, halophytes, physiological integration, rhizomes, salinity, stolons

## Abstract

Plant modularity-related traits are important ecological determinants of vegetation composition, dynamics, and resilience. While simple changes in plant biomass resulting from salt treatments are usually considered a sufficient indicator for resistance vs. susceptibility to salinity, plants with a clonal growth pattern show complex responses to changes in environmental conditions. Due to physiological integration, clonal plants often have adaptive advantages in highly heterogeneous or disturbed habitats. Although halophytes native to various heterogeneous habitats have been extensively studied, no special attention has been paid to the peculiarities of salt tolerance mechanisms of clonal halophytes. Therefore, the aim of the present review is to identify probable and possible halophytic plant species belonging to different types of clonal growth and to analyze available scientific information on responses to salinity in these species. Examples, including halophytes with different types of clonal growth, will be analyzed, such as based on differences in the degree of physiological integration, ramet persistence, rate of clonal expansion, salinity-induced clonality, etc.

## 1. Introduction

Plant modularity-related traits are important ecological determinants of vegetation composition, dynamics, and resilience [1]. Clonality in plants represents an especially advanced case of modularity, where, at least in theory, an individual module (ramet) can become functionally independent from a parent organism (genet), realizing the process of vegetative reproduction [2]. However, from a functional point of view, more important are those cases when the newly formed ramets maintain a connection with the parent organism throughout the entire season or even several seasons. Similar to non-clonal modular organisms, which use the exchange of resources and signals between different modules, ramets can benefit from association with other ramets. Moreover, similar to functional differences between different organs, individual ramets can become partially specialized for particular functions, such as resource acquisition (foraging), photosynthesis, overwintering, or storage.

The morphology and life cycle of the particular clonal plant species depend on the type of its clonal growth organ (CGO). According to the recent classification system, there are seven major groups of CGOs: stolons, two types of rhizomes (epigeogenous and hypogeogenous), stem tubers, root tubers, bud-bearing roots, and bulbs [3]. Most importantly, each type of clonal growth (manifested by a presence of a specific CGO) has a specific developmental pattern(s) [3]. Moreover, there are significant variations in the degree of clonal integration, clone persistence, etc., even within species having the same general type of clonal growth organ. Therefore, developmental and growth responses to changing environmental conditions of clonal species cannot be predicted only on the basis of biomass measurements.

Recently, scientific interest in clonal plant biology has been renowned through a series of publications arguing for the necessity of studies integrating ecology and evolution of clonal plants with functional aspects and highlighting current key scientific questions and problems in this direction [1,3,4,5,6,7,8,9,10,11,12,13]. It is indeed surprising that the ability of plants to grow clonally has been overlooked for decades; especially, given the wide distribution of clonal species. Thus, in Central European flora, about 66% of species have some type of clonal growth [3]. In several habitats, the proportion of clonal species is even higher. For example, in a grazed, wet Atlantic coastal meadow, 81% of species were clonal and contributed to 97.7% of the vegetation cover [14]. 

Clonality can be regarded as an adaptive life history feature of plants living in highly heterogeneous habitats, such as the ones in alpine, coastal, or wetland ecosystems. In particular, the abundance of clonal life forms in coastal habitats, both dune- and wetland-related, has been emphasized earlier [15]. For example, when coastal habitats, associated with strandlines, are considered, it is stressed that strandline “colonist species” (plants being able to establish in these habitats) in the majority are “clonal perennials with extensive rhizomatous or stoloniferous growth” [16]. As salinity is one of the major factors controlling species distribution and abundance in coastal habitats [17,18,19], it would be logical to ask how salinity affects aspects of clonal growth in coastal species. Surprisingly, the presence of clonal growth characteristics is largely a neglected aspect in plant salinity tolerance studies, and so far, no study has focused in particular on halophytic clonal plant species. Although halophytes native to various heterogeneous habitats have been extensively studied, no special attention has been paid to the peculiarities of salt tolerance mechanisms of clonal halophytes. While simple changes in plant biomass resulting from salt treatments are usually considered a sufficient indicator for resistance vs. susceptibility to salinity, plants with a clonal growth pattern show complex responses to changes in environmental conditions [20]. Due to physiological integration, clonal plants often have adaptive advantages in highly heterogeneous or disturbed habitats [21]. Therefore, it would be important to find out if there are clonal halophyte species for which salinity promotes clonal growth and/or affects aspects of physiological integration. Although clonal halophytes and their response to salinity have been studied relatively and often under controlled conditions, the results obtained usually do not allow for the evaluation of clonal growth changes because the experiments were only short-term or only non-clonality-related traits were evaluated. However, useful information on possible changes in clonality due to heterogeneity of salinity can be obtained from studies under natural conditions.

In contrast to plant clonality, which can be defined by specific morphological features, salt tolerance is more difficult to assess. When describing the salt tolerance of wild plant species, the term “halophyte” is usually used. The term has a relatively long and sometimes controversial history, as analyzed recently [22]. The most accepted halophyte definition today might be the one suggested by T.J. Flowers and T.D. Colmer, namely, that halophytes are “plants that survive to reproduce in environments where the salt concentration is around 200 mM NaCl or more” [23]. Since salt tolerance is determined both under experimentally controlled conditions and based on soil measurements in natural habitats, the following relationships should also be understood regarding the salinity level used to define halophytes. From the point of soil science and ecology, soil salinity is evaluated according to the electrical conductivity of saturated soil extract (EC_e_) in dS m^−1^ [24]. According to the common criteria, slightly saline soils have EC_e_ 2.1 to 4.0 dS m^−1^, moderately saline 4.0 to 8.0 dS m^−1^, strongly saline 8.1 to 16.0 dS m^−1^, and very saline >16.0 dS m^−1^ [25]. In this respect, the threshold salinity value for halophyte definition, 200 mM NaCl, roughly corresponds to 20 dS m^−1^ [26].

The aim of the present review was to identify probable and possible halophytic plant species belonging to different types of clonal growth and to analyze available scientific information on responses to salinity in these species. To achieve this aim, first, the information that is available in a dataset from the CLO-PLA database supplementing the paper on the evolution of clonal growth forms in angiosperms [3] was compared with the data available in the eHALOPH database (V4.65, https://ehaloph.uc.pt, last visited 15 February 2023 [26]. Additional data were taken from the supplement to the paper on ecological indicator and trait values for Swedish vascular plants [27] and the list of plant species found in salt-affected coastal habitats of the Baltic Sea [28,29]. As a result, a working list of potential clonal halophytes characteristic for Northeastern Europe (Baltic Sea region) was prepared (Table 1). Halophyte species with tubers and bulbs as the only CGO type were not included in the list. These species were indicated as non-clonal due to their low values of lateral spread and multiplication rate [3]. Second, available information on salinity-related aspects of these species was searched in databases of scientific literature. The list of Central European inland salt marsh species was used to obtain values of maximum soil EC_e_ where the particular species was found [30]. If necessary, information was supplemented for taxonomically or morphologically related species from other regions in order to achieve a more substantial degree of generalization of the analysis of clonal halophyte species. Third, where possible, species descriptions were supplemented with original photographs to illustrate the diversity of their habitats. The nomenclature used follows that of The World Flora Online (http://www.worldfloraonline.org, last visited 5 March 2023). In the cases when information about the plant has been published under a different name, corresponding synonyms have been added.

The amount of information available for different species regarding their clonality, salt tolerance, and in particular, changes in clonality in response to salt, varied widely. Some species had successful literature reviews that were not repeated in detail here. For some species, there was very little information on their clonal growth pattern, response to salinity, or both. Therefore, it was possible to give only a general description and indicate the directions of the necessary missing studies. Potential clonal halophyte species (including both species defined as halophytes as well as species with salt tolerant ecotypes) were grouped according to the type of their dominant CGO in the sense of Herben, Klimešová (2020) [3].

## 2. Halophyte Species with Stolons

### 2.1. General Aspects

It appears that stolons, as the dominant CGO among halophytic species characteristic of the Baltic Sea region, are relatively rare. There were only five species in this group: two grasses (Poaceae; *Agrostis stolonifera* and *Puccinellia maritima*), two legumes (Leguminosae; *Trifolium fragiferum* and *Trifolium repens*), and *Potentilla anserina* (Rosaceae). In addition, the South African stoloniferous species *Carpobrotus edulis* was included in the analysis due to the presence of biological characteristics important for the invasiveness of the species. Besides, *Calystegia sepium* is indicated as having stolons as the dominant type of CGO [3]; however, it was analyzed under species with hypogeogenous rhizomes due to their dominant importance in the lateral clonal spread of the species. One more species, *Hydrocotyle vulgaris,* is indicated as having epigeogenous rhizomes as the dominant CGO type [3], and was included in that group; however, in natural conditions, it most likely has stolons as the dominant type.

### 2.2. Agrostis stolonifera

**Figure 1:**
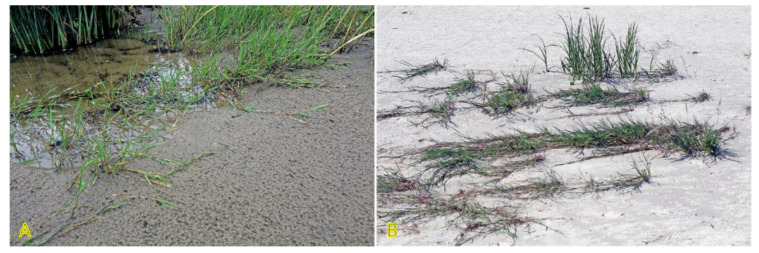
*Agrostis stolonifera* plants on a flooded wet sandy beach together with *Bolboschoenus maritimus* in Lielupe, Jūrmala, Latvia (**A**) and a relatively dry sandy beach together with *Schoenoplectus tabernaemontani* in Ainaži, Latvia (**B**).

*Agrostis stolonifera* can be found in different successional zones of the beach plain with various flooding–salinity regimes, even being dominant in some of them [31]. In the Baltic coastal wetlands, *A. stolonifera* is the most abundant species on the lower shore, being abundant also on the upper shore and present in open pioneer communities [19]. With a frequency of 33%, the species occurs in Central European inland salt marshes at maximum soil salinity EC_e_ 118.0 dS m^−1^ [30]. Adaptation to a wide range of substrate moistures, from soil flooding to drought-prone conditions, is characteristic for the species (Figure 1). *A. stolonifera* plants produce long stolons with an ability to form both tillers and roots at nodes. The species has been characterized as an “above-ground splitter” [14]. Aspects of clonality of *A. stolonifera* have been studied with respect to heterogeneous mineral nutrient availability, and it was found that rooted ramets are relatively independent with respect to mineral nutrient acquisition and use, pointing to a relatively low level of physiological integration [32].

The species has been listed among grass halophytes [33] and it has been evaluated as “favoured by moderate salinity, but not restricted to such habitats” in Sweden [27]. Practically oriented studies on the salinity tolerance of *A. stolonifera* have been performed with respect to turfgrass resilience [34,35]. In addition, *A. stolonifera* has been used as a model species to demonstrate the regulatory role of microRNAs in response to salt stress using transgenic plants [36].

The existence of ecotypes of *A. stolonifera* with different salinity tolerances has been established in numerous studies [37,38,39,40,41]. Tolerance to NaCl and CaCl_2_ of different *A. stolonifera* ecotypes were identical, but MgCl_2_ exhibited more negative effects [39]. Only the salt marsh clone of *A. stolonifera* had pronounced tolerance to MgCl_2_ [42]. Among anions, biomass accumulation was most sensitive to carbonate, followed by sulfate and chloride [43]. The effect of the type of salt treatment (soil drench vs foliage spray) was compared using plants from sea cliffs and inland populations of *A. stolonifera*, and it was found that both populations were equally sensitive to soil NaCl but the sea cliff population was more tolerant to salt spray in comparison to the inland population [44]. Variations in clonal morphology of plants in different native habitats have been noted [45]. Thus, plants in conditions of inland fertilized meadow produced a low number of thick and long stolons, a large number of thin and short stolons were produced by plants in the salt marsh site, while plants growing in nutrient-limited sand dune conditions developed a low number of thin and long stolons.

Salt tolerance of *A. stolonifera* increased for plants from inland habitat (87.4% growth inhibition at 0.2 M NaCl) to spray zone habitat (51.5% growth inhibition) and further to salt marsh habitat (27.1% growth inhibition) [38]. Moreover, plants from salt marsh habitat had pronounced waterlogging tolerance. When the interactive effect of inundation and salinity was tested in controlled conditions, it appeared that soil waterlogging and flooding both stimulated stolon growth, but shoot biomass did not increase [46]. However, when inundation was performed with seawater, stolon growth was not stimulated but shoot biomass increased instead. In a study with plants from three groups of *A. stolonifera* populations (from saline maritime habitats with occasional inundation by seawater, from non-saline maritime habitats subject to salt spray from non-saline inland habitats), it was found that plants from non-saline soils were more negatively affected by NaCl during cultivation in a controlled condition [37]. However, plants from populations native to saline soils accumulated less Na^+^ in shoots under salinity (<11 g kg^−1^) in comparison to that in plants from non-saline populations (up to 28 g kg^−1^). Similarly, anions of Na^+^ salts that had a more inhibitory effect on biomass accumulation resulted in higher Na^+^ accumulation in shoots [43]. These observations are fully consistent with the hypothesis that salinity tolerance of monocotyledonous halophytes, including grass species, is associated with salt exclusion strategy [23]. In natural conditions of salt-affected coastal habitats, *A. stolonifera* accumulated <10 g Na^+^ kg^−1^ in leaves [29,37], controlling tissue electrical conductivity mostly by the means of changes in K^+^ concentration [29].

Unfortunately, no study so far had assessed the effect of salinity on clonal growth characteristics of *A. stolonifera* or, at least, the changes in biomass distribution among different plant parts as a result of salinity. However, the role of clonal dispersal for *A. stolonifera* was tested with respect to flooding, and it was concluded that clonality gives no advantage to biomass accumulation in these conditions, but it increases the general competitive ability of individuals [47].

Morphologically similar stoloniferous grass species, *Paspalum paspaloides*, benefited from clonal integration during expansion in saline aquatic habitats [48]. Most importantly, Na^+^ was preferentially accumulated in the apical part of stolons (up to 60 g kg^−1^ dry mass (DM)), followed by leaves in the apical part (up to 22 g kg^−1^ DM), but accumulation was restricted in roots as well as in basal parts located in non-saline soil.

### 2.3. Carpobrotus edulis

*Carpobrotus edulis* is a stoloniferous coastal species native to South Africa, becoming invasive or naturalized in many countries mainly in Mediterranean-type climate conditions [49]. The biology of *C. edulis* and taxonomically related species has been reviewed in detail relatively recently [49,50]; therefore, only some aspects of the clonal and halophytic nature of the species will be briefly mentioned here. It needs to be emphasized that both features, clonality and halophytism, are important for higher competitive ability of *C. edulis* in comparison to other coastal species. First, the species can benefit from a high level of physiological integration not only in heterogeneous but also in homogeneous conditions, and this involves the participation of stolons both for clonal expansion as well as storage organs [51]. Apical ramets of colonizing *C. edulis* plants clearly benefit from clonal integration [52]. Second, at the range of salinity similar to that present in the habitats with *C. edulis*, plant growth was significantly stimulated, including stimulation of the formation of new shoots [53]. It was suggested that trans-generational effects need to be further assessed in order to better understand the extremely high invasion potential of *C. edulis* [49].

### 2.4. Potentilla anserina

**Figure 2:**
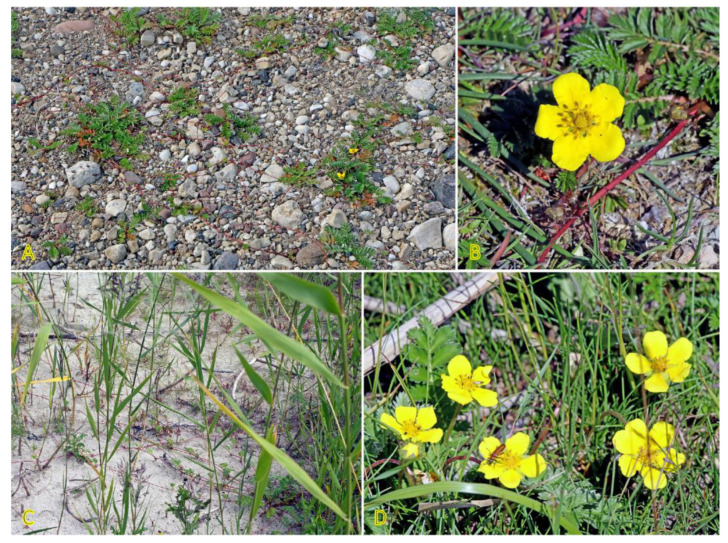
*Potentilla anserina* plants on a gravel beach on the island of Saaremaa, Estonia (**A**), coastal meadow in Mērsrags, Latvia (**B**,**D**), and sandy beach in Mērsrags, Latvia (**C**).

*Potentilla anserina* is one of the most widely studied stoloniferous species with respect to clone demographics, clonal integration, and effects of environmental constraints. The species is relatively shade-intolerant, being associated mostly with open wet meadows and sandy habitats along rivers and on beaches [54,55]. *P. anserina* plants are frequently found in salt-affected habitats (Figure 2). Thus, the population of *P. anserina* from a seashore meadow of the Baltic Sea has been used for a series of studies by O. Eriksson in the 1980s [56,57,58,59,60]. With a frequency of 25%, the species occurs in Central European inland salt marshes at maximum soil salinity of EC_e_ 22.2 dS m^−1^ [30]. In South Korea, the distribution of *P. anserina* is limited to coastal lagoons, where it forms an association with another clonal halophyte species, *Lysimachia maritima*, growing in moderately saline soils containing 1.7–1.9 g Na^+^ kg^−1^ [61]. While not being listed in the eHALOPH database, in Sweden, *P. anserina* is characterized as “favoured by moderate salinity, but not restricted to such habitats” (salinity tolerance level 3 out of 5) [27]. However, no studies so far have directly assessed the salinity tolerance of the species. Existence of accessions with different degrees of salinity tolerance can be predicted. Moderate but highly variable Na^+^ accumulation potential in leaves of *P. anserina* was evident in different salt-affected coastal habitats of the Baltic Sea, and existence of two clear subtypes was seen with respect to preferential accumulation of either more K^+^ or Na^+^ [29].

Plants form leaf rosettes and new stolons in spring. Both stolons and flowers are developed from rosette leaf axils, resulting in a trade-off between generative and vegetative reproduction [60]. Stolons are up to 1 m long on average, usually only one per plant, with 1.7–3.0 daughter ramets; however, maximum clonal growth in favorable years can produce 10 stolons with 50 ramets per genet [57]. As a result, *P. anserina* has a prominent capacity for vegetative spread, forming dominant stands with 2000 ramets per m^2^ in coastal meadows [58]. However, plants established from seeds form flowers or stolons only after five years. All rooted ramets form short rhizomes acting as overwintering structures when they become independent in autumn after the dieback of stolons [60].

These features of clonal physiology resulting in high morphological plasticity give the plant adaptive advantages in different situations of environmental heterogeneity, both in the context of resources and conditions. Modular plasticity of *P. anserina* has been studied with respect to light and nutrient variability [62,63,64], heavy metals [65,66], sand burial [67], and soil moisture [68]. It can be generalized from the above studies that clonal integration and functional specialization of ramets are indeed prominent adaptive characteristics in order to cope with environmental disturbances as well as resource heterogeneity. However, relative costs of clonal propagation and sexual reproduction are modified by environmental factors.

The large potential practical importance of *P. anserina* is mainly associated with its pharmacological properties [69,70]. On the other hand, starchy roots of *P. anserina* have been traditionally used for food in Iceland and other Nordic regions [71] as well as in Mongolia [72]. Experimental identification of salt-tolerant ecotypes of *P. anserina* is an important aspect in the context of further practical use of the species.

### 2.5. Puccinellia spp.

**Figure 3:**
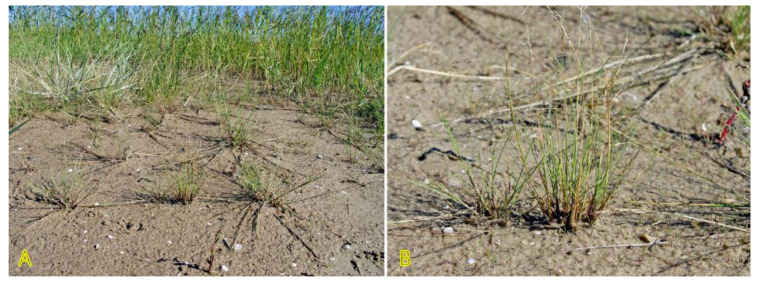
*Puccinellia maritima* plants on a wet sandy beach together with *Leymus arenarius* and *Phragmites australis* (**A**), genet with characteristic clonal architecture, and (**B**) in Lielupe, Jūrmala, Latvia.

Species of the genus *Puccinellia* are generally known as “alkali grasses” because of their high tolerance to alkaline salinity [73]. *Puccinellia maritima* is often described as a dominant species of the lower stabilized zone of Northwestern European salt marshes [74], but it occurs also in the pioneer zone with the start of sediment accumulation [75]. In addition, *P. maritima* is one of the first colonizing species during the formation of vegetation during the establishment of dune ridges [76]. The plants trap sand resulting in the appearance of low hillocks as a basis for the formation of dune ridges. However, in the Baltic region, *P. maritima* and other species of the genus, such as *Puccinellia distans*, are more often found on dune slacks and permanently wet, undisturbed beaches with low-intensity of sand accretion, such as spring-affected sandy beaches (Figure 3). Both species in Sweden are characterized as “favoured by moderate salinity, but not restricted to such habitats” (indicator level 3 out of 5) [27]. Both *P. distans* and *P. maritima* are included in the clonal plant database, where *P. distans* is characterized by low clonal spread by means of epigeogenous rhizomes (0.5 cm per year) in contrast to intensively spreading stoloniferous *P. maritima* (20 cm per year) [3]. In the northern part of the distribution range, *P. maritima* mainly propagates vegetatively through the formation of adventitious roots at the nodes of stolons [73]. Both *P. distans* and *P. maritima* are included in the eHALOPH database, but only *P. distans* is mentioned among grass halophytes [33].

Not much experimental evidence is available on the salinity tolerance of *P. maritima* or *P. distans*. In hydroponic conditions, the survival rate of *P. maritima* was 55.0% at 10 g L^−1^ NaCl, in comparison to only 1.6% at 30 g L^−1^ NaCl, and biomass accumulation was inhibited by 77.5 and 97.5%, respectively [73]. *P. distans* plants were able to sustain full growth at 200 mmol NaCl and to tolerate irrigation with 600 mmol NaCl, with only a 37% reduction of shoot biomass [77]. Another species of the genus, *Puccinellia tenuiflora*, an important forage species, has emerged as a model plant in studies of the salinity and alkalinity tolerance of halophytic grasses [78,79]. Growth of *P. tenuiflora* was not negatively affected in hydroponics with 300 mM NaCl or NaHCO_3_ [80].

### 2.6. Trifolium spp.

**Figure 4:**
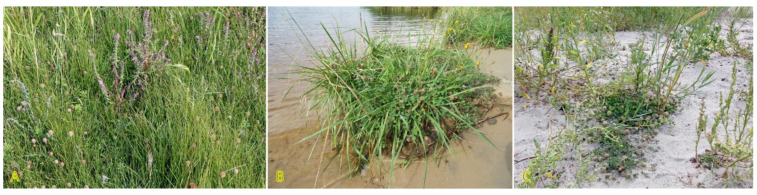
*Trifolium fragiferum* plants on a wet coastal meadow together with *Odonites vulgaris*, *Potentilla anserina,* etc., on the island of Kihnu, Estonia (**A**), on the shore of river Buļļupe, Buļļusala, Rīga, Latvia (**B**), and on the sandy beach in Varbla, Estonia (**C**).

Two *Trifolium* species with a stoloniferous clonal growth type, *Trifolium fragiferum* and *Trifolium repens*, can be found in coastal habitats of the Baltic region, often coexisting in salt-affected wet meadows [81]. Besides, *T. fragiferum* can be found on wet habitats along freshwater shores as well as on relatively dry coastal grasslands and even on sandy beaches (Figure 4) [82]. *T. fragiferum* is included in the eHALOPH database, but *T. repens* is not. However, the salinity tolerance of *T. fragiferum* in Sweden has been indicated as 3, in comparison to that of 2 in *T. repens* [27]. Due to the presence of *T. fragiferum* as a component of salt marsh vegetation, the species has been designated as an obligatory mesohydrohalophile [83]. Both species occur in Central European inland salt marshes with a frequency of 11 and 6%, for *T. fragiferum* and *T. repens*, respectively [30].

Screening of a large number of *T. fragiferum* accessions and cultivars revealed that wide genetic diversity is present within the species with respect to salinity tolerance [84]. Detailed studies of salinity tolerance of various *T. fragiferum* accessions from habitats with different salinity levels propagated by seed have been performed recently in controlled conditions [85]. Plants were able to grow in a substrate with 217 mmol L^−1^ NaCl with no visible signs of toxicity, but their growth and development (number of stolons, stolon elongation, number of leaves, biomass) were significantly suppressed already at 87 mmol L^−1^ NaCl. Significant differences were found between the accessions in salinity responses with respect to inhibition of biomass accumulation as well as tissue water content. However, while *T. fragiferum* accession native to the most saline soil indeed showed the highest relative tolerance to salinity, these plants had the lowest shoot biomass already in control conditions. Consequently, other accessions from moderately saline conditions showed higher biomass in saline conditions.

For clonal *Trifolium* species, biomass allocation to stolons can be viewed as an indication of investment in clonal growth. However, the establishment of ramets capable of overwintering depends on the rooting ability of stolons as well as necessary environmental conditions (mostly humidity). On the background of different degrees of inhibition of biomass accumulation in various *T. fragiferum* genotypes, the relative proportion of allocation to stolons did not change much, but allocation to generative structures increased with increasing salinity [85]. Consequently, at increasing substrate salinity, *T. fragiferum* plants invest in flower development at the expense of leaf biomass, with no effect on relative allocation to clonal growth. This is in contrast to the study showing that clonal growth is a priority investment direction compared to sexual reproduction for both *T. fragiferum* and *T. repens* [86]. It needs to be emphasized that due to the specific architecture of stoloniferous *Trifolium* species, stolon branching and flowering represent two mutually exclusive events in the individual development of ramets.

At moderate salinity (up to 87 mmol L^−1^ NaCl or soil electrical conductivity 3.0 mS cm^−1^), different accessions of *T. fragiferum* accumulated identical levels of Na^+^ in leaf blades and petioles (5.5–6.5 and 15.0–20.0 g kg^−1^ DM), but at high salinity, genotype-specific differences were evident, as accessions from the most saline sites accumulated more Na^+^, reaching 25 and 55 g kg^−1^, respectively [85]. However, an increase in Na^+^ in roots with increasing substrate NaCl had a saturable character already at low salinity, and reached only 5.5–8.0 g kg^−1^ DM. Similar Na^+^ accumulation potential was seen for roots.

Transgenerational effects were studied in *T. repens*, and it was concluded that epigenetic change by water shortage was evident across several clonal offspring generations [87]. Most importantly, transgenerational effects were highly genotype- as well as environmental-factor-specific (heavy metals, salinity, water shortage, and shade). In this respect, results from the study with *T. repens* “ecotypes” showing that clonally propagated plants from different natural soil salinity gradient zones respond differentially to flooding by seawater, need to be addressed with caution [88]. As expected, plants originating from more saline zones showed fewer negative effects from salinity in comparison to the ones from less saline zones, but it was concluded that the response was due to ecotype specificity, and no possibility of epigenetic regulation was considered.

Practical interest in *T. fragiferum* has been associated with the fact that the species can be used as a component in temperate perennial grasslands and pastures in problematic agroecological conditions, such as high salinity, alkalinity, and soil waterlogging [89]. Wild accessions of *T. fragiferum*, especially from the northern border of the distribution range, have a high tolerance against soil flooding, cutting, and trampling, showing them as a promising source of abiotic stress resistance genes for further breeding [90].

## 3. Halophyte Species with Epigeogenous Rhizomes

### 3.1. General Aspects

The explorative aspect of clonal plants with branching epigeogenous rhizomes and clear phalanx growth strategy is very limited [91]. However, while these species have a low potential for vegetative spread, they are still able to occupy new sites by establishing individuals from rhizome fragments that have separated from the mother plants after storms and are carried to new locations by currents.

Four out of five species from this group were typical phalanx-type species: three monocotyledonous species (*Iris pseduacorus* from Iridaceae, *Triglochin maritima* and *Triglochin palustris* from Jancaginaceae) and Compositae species *Tripolium pannonicum*. However, *Hydrocotyle vulgaris* (Araliaceae), included in this group, represent species with a guerilla clonal growth strategy.

### 3.2. Hydrocotyle spp.

**Figure 5:**
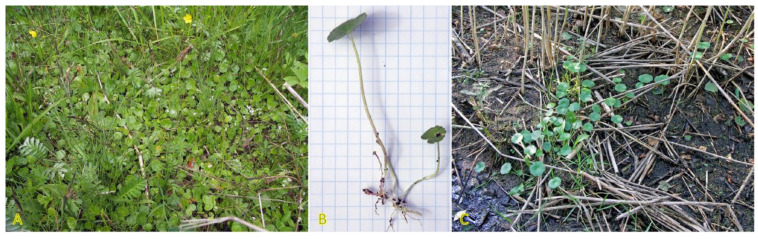
*Hydrocotyle vulgaris* plants dominating in a wet coastal meadow in Mērsrags, Latvia (**A**), the apical fragment of the shoot (**B**), foraging plant on the shore of Lake Būšnieks, near Ventspils, and Latvia (**C**).

Clonal species of the genus *Hydrocotyle* are characterized by high morphological plasticity resulting from changes in environmental conditions [92]. *Hydrocotyle vulgaris* is a perennial species with long plagiotropic branching shoots and stolons, which can be located also in the upper layer of the soil, formally making this structure an epigeogenous rhizome. However, unlike typical species with epigeogenous rhizomes, the clonal spread of *H. vulgaris* is prominent, up to 18.4 cm per year; however, spacer distances are rather short [3]. At each node, a single leaf and adventitious roots are present, often together with a flower. Due to intense branching and short spacer distances, *H. vulgaris* plants often dominate their habitats (Figure 5).

Physiological and ecological aspects of clonal integration in *H. vulgaris* have been extensively studied, recently mostly in conjunction with the high potential invasiveness of the species in China. A very high level of clonal integration seems to be associated with the expressed invasive character of the species, especially benefiting in conditions of heterogeneous water supply [93] and nutrient heterogeneity [94,95], as well as for increasing intraspecific competitiveness [96]. Although, it was not possible to experimentally prove the hypothesis that the invasiveness of *H. vulgaris* is directly related to the phenotypic plasticity of its individuals, as the results were inconsistent [97]. However, from a clearly practical point, it was shown that nutrient enrichment reduced the competitive ability of *H. vulgaris* in native communities [98].

In the Baltic region, *H. vulgaris* can be found in wet coastal and shore meadows, both within relatively open, low-herb vegetation as well as in full shade under tree canopies [99]. *H. vulgaris* is not included in the eHALOPH database, and the salinity tolerance of the species is indicated to be only 1 out of 5 (“not salt tolerant, avoiding also weakly saline conditions”) according to the list of ecological indicators in Sweden [27]. However, the species has been found in saline habitats, wet coastal meadows with fluctuating soil salinity and with soil Na^+^ concentration reaching 3 g L^−1^ [99]. Light conditions significantly affected the response of *H. vulgaris* plants to salinity. In low light conditions, similar to these under canopy shade in natural conditions, low NaCl (132 mmol substrate salinity) stimulated the growth of both leaves as well as primary stolons [100]. However, the growth of secondary stolons was stimulated in low light by low and moderate salinity (341 mmol), and in moderate light by low salinity. All growth parameters were decreased by low and moderate salinity in high-light conditions. Consequently, salinity episodes for plants growing in canopy shade stimulated the clonal expansion of *H. vulgaris*. It still remains to be checked whether clonal integration has any adaptive meaning in conditions of fluctuating soil salinity for *H. vulgaris*; especially, on the background of different light regimes.

In natural conditions of saline coastal habitat, leaves of *H. vulgaris* accumulated up to 80 g Na^+^ kg^−1^ DM at the beginning of the season decreasing to 35–40 g kg^−1^ later [100]. In controlled conditions, maximum Na^+^ accumulation for plants cultivated at 8 g Na^+^ L^−1^ substrate was 35 g kg^−1^ in leaf blades, 28 g kg^−1^ in leaf petioles, and 6 g kg^−1^ in stolons [101], showing typical characteristics of salt-accumulating species.

The taxonomically and morphologically similar species, *Hydrocotyle bonariensis*, colonizes coastal dunes in the Southeastern United States, but can be found also in adjacent salt marshes [102]. *H. bonariensis* form extremely large genets with thousands of connected ramets occupying over 40 m^2^ of dune surface [103]. A high level of physiological integration can be seen with respect to resource acquisition and translocation [103,104] and it also gives an ability to avoid patches of grass [105] as well as promotes resilience to grazing [106]. Most importantly in the context of the present review, clonal integration allowed for undisturbed growth of *H. bonariensis* in conditions of spatially heterogeneous salinity, when ramets located in soil with increased salinity were supported by ramets located in non-saline spots, with no negative effects on growth [102]. In contrast, plants in homogenous salinity conditions or separated ramets without the benefit of integration showed little biomass accumulation and high mortality.

### 3.3. Iris spp.

*Iris pseudacorus* can be relatively frequently found in British salt marshes [107] as well as in salt-affected coastal habitats of the Baltic region [29], but in general, the species is thought to not be salt tolerant [27]. Clonal spreading occurs radially, forming large genets, but rhizome fragmentation occurs after about ten years, and rhizome fragments can be transported by water and established in new sites [107]. Leaves make up only a small part of total biomass, about 1% [108]. Not much experimental evidence is available on the salinity tolerance of *I. pseudacorus*, but it was noted that plants can survive in soil with 10 g kg^−1^ NaCl [107].

*Iris hexagona*, a North American species, is considered to be a glycophyte, but it is frequently found in intermediate-brackish coastal wetlands [109]. No intraspecific variability in salinity tolerance was evident between *I. hexagona* poplayions from freshwater vs brackish wetlands in spite of clear genetic differences [110]. Even low salinity (irrigation with 4 mg L^−1^ NaCl) reduced below-ground biomass by 50%, but the number of produced seeds increased twice [111].

In freshwater Louisiana wetlands, *I. hexagona* co-occurs with introduced and invasive species *I. pseudacorus* [112]. Initially, it was suggested that *I. pseudacorus* can outcompete *I. hexagona* because of the higher salinity tolerance of the former. However, it appeared that *I. pseudacorus* has a competitive advantage over *I. hexagona* regardless of the actual salinity due to more vigorous clonal growth of the introduced species [108]. Salinity had a similar pronounced negative effect on the growth of both species.

Another rhizomatous species of the genus, *Iris halophila*, native to saline-alkaline habitats, has been considered tolerant to salinity [113]. In conditions of hydroponics, root and shoot growth were not negatively affected by 150 mM NaCl, but Na^+^ was preferentially accumulated in roots (8 mmol g^−1^ DM) in comparison to leaves (3 mmol g^−1^ DM).

### 3.4. Triglochin spp.

**Figure 6:**
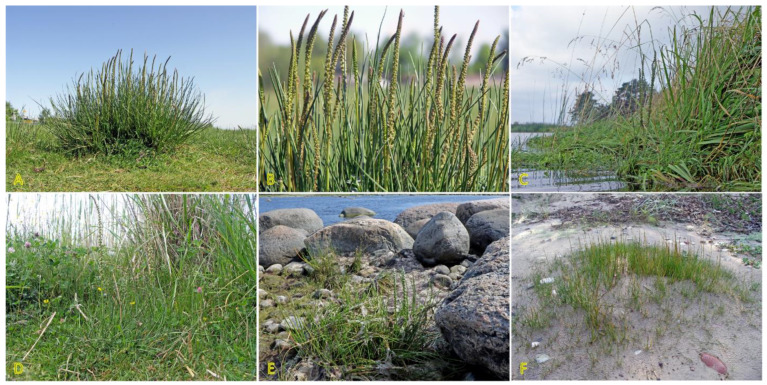
*Triglochin maritima* plants on a salt-affected meadow in Liepāja, Latvia (**A**), detail of inflorescences (**B**), on saline shores of river Buļļupe, Rīga, Latvia (**C**), on a coastal meadow in Mērsrags, Latvia (**D**), on a stony beach in Matsi-Sömeri, Estonia (**E**). *Triglochin palustris* on a spring-affected wet beach in Liepene, Latvia (**F**).

Two rhizomatous species of the genus *Triglochin* are important in the context of the present review: *Triglochin maritima* and *Triglochin palustris*. According to molecular phylogenic analysis, *T. maritima* and *T. palustris* form a complex consisting of *T. palustris*, Eurasian *T. maritima*, and American *T. maritima* clades [114]. In contrast to the European Atlantic populations, coastal populations of *T. maritima* of the North Sea and the Baltic Sea originated from inland populations during postglacial colonization [115]. Both species are listed in the eHALOPH database, and have been evaluated as “competitive only under moderate–high salinity” and “moderately salt tolerant, but preferring non-saline conditions”, respectively, according to the ecological indicator values in Sweden [27]. *T. maritima* is an obligate wetland species and has been widely used as indicator species of saline habitats. It can be found in coastal salt marshes, rocky shores with grass vegetation, wet beaches with perennial vegetation, and wet salt-affected meadows (Figure 6) [116]. The species has often been found in Central European inland salt marshes (frequency 28%) [30].

*T. palustris* has not been specifically associated with typical coastal habitats in the Baltic Region [27], but it appears on permanently wet sandy beaches (Figure 6). In Canada, *T. palustris* can be found in brackish conditions on soils rich in Mg and Ca [117]. Interestingly, *T. palustris* has been reported as a roadside halophyte [118].

The clonal spread of *T. mariitima* is relatively limited, as it is only 0.5 cm per year [3], but due to the longevity of genets, the diameter of clumps can reach 2 m [116]. The clonal spread rate of *T. palustris* is higher (6.8 cm per year) [3], and it can form scattered wider genets (Figure 6F). Elevated rings of *T. maritima* due to production of shallow roots in New England salt marshes ameliorate waterlogging and contribute to increased species diversity [119].

In non-limiting nutrient conditions, treatment with 340 mol L^−1^ NaCl had no effect on the growth of *T. maritima*, but it was inhibited by waterlogging with saline solution [120]. *T. maritima* plants seem to be adapted to soils with highly variable content of plant-available nutrients. Especially high variation among different samples of native soil was evident for potassium, sulphur, iron, manganese, zinc, copper, and boron [121]. Characteristically low nitrogen and potassium concentration was accompanied by a high and extremely high concentration of other nutrients as well as sodium and chloride (in a range 0.2–1.9 and 0.3–2.2 g L^−1^), respectively. The concentration of both iron and magnesium was in a zone of potential toxicity, reaching 4.0 and 2.5 g L^−1^, respectively. In controlled conditions, *T. maritima* plants did not respond negatively to treatment with Mg and Fe (4 g kg^−1^) in a form of sulphate, but treatment with Na_2_SO_4_ at the same amount stimulated both leaf and root growth [122].

Among monocotyledonous halophytes, *T. maritima* is characterized as an exceptional Na^+^ accumulator. In natural conditions of salt-affected coastal habitats, Na^+^ concentration in leaves of *T. maritima* varied from 25 to 60 g kg^−1^ DM, and it was among a few halophytic species controlling tissue electrical conductivity by means of changes in Na^+^ concentration [29]. In controlled conditions at moderate substrate salinity, only 13 g Na^+^ kg^−1^ DM was accumulated, and it decreased to 7 g kg^−1^ in presence of 14 g Ca^2+^ kg^−1^ [122]. Interestingly, in non-saline conditions, leaves of *T. maritima* accumulated 21.6–25.5 g Na^+^ kg^−1^ DM, but at 340 mmol L^−1^ NaCl, the accumulation increased to 32.4–33.4 g kg^−1^ [120].

### 3.5. Tripolium pannonicum

**Figure 7:**
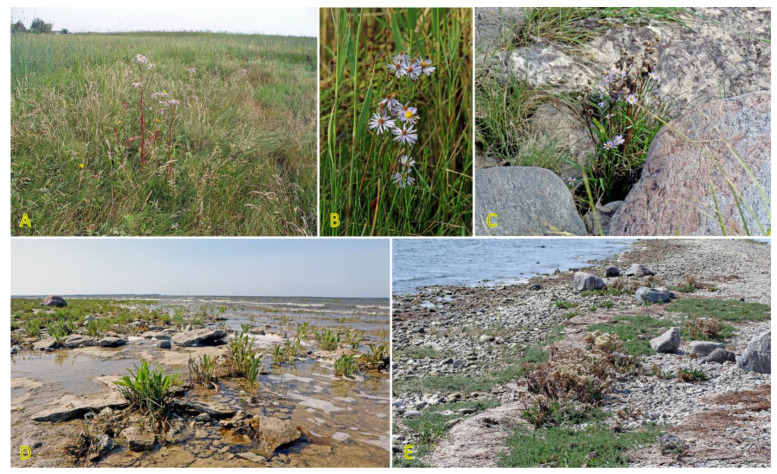
*Tripolium pannonicum* plants in a salt-affected wet meadow in Liepāja, Latvia (**A**), a coastal wetland together with *Phragmites australis* on the island of Kihnu, Estonia (**B**), on a stony beach in Matsi-Sömeri, Estonia (**C**), on a rocky beach on the island of Saaremaa, Estonia (**D**), on a pebble beach together with *Lysimachia maritima* on the island of Hiiumaa, Estonia (**E**).

Similar to *Triglochin maritima*, the clonal spread of *Tripolium pannonicum* (syn. *Aster tripolium*) is relatively limited, only 0.5 cm per year [3]. No studies so far have experimentally assessed the potential role of clonal growth in the spread of *T. pannonicum* or the effect of environmental factors, including salinity, on clonal growth. However, distribution of detached rhizome fragments by water is highly possible; especially, in habitats with limited generative reproduction. In the Baltic region, the species can be found in a variety of wet coastal habitats (Figure 7). *T. pannonicum* is often found in Central European inland salt marshes (frequency 31%) at maximum soil salinity EC_e_ 118.0 dS m^−1^ [30].

*T. pannonicum* is a typical salt-accumulating hygrohalophyte species, characterized by relatively high salinity tolerance with an ecological indicator value of 4 out of 5 [27]. In natural conditions, *T. pannonicum* accumulates a high level of Na^+^ in leaves (25–75 g kg^−1^ DM), and tissue electrical conductivity is regulated mainly by changes in Na^+^ on the background of relatively low K^+^ level [29]. Numerous studies in controlled conditions have assessed different aspects of the salinity tolerance of *T. pannonicum* [101,123,124,125,126,127,128,129,130].

## 4. Halophyte Species with Hypogeogenous Rhizomes

### 4.1. General Aspects

Halophytes with hyogeogenous rhizomes as a dominant GCO formed the largest group, consisting of 25 species. Among them, 14 were monocotyledonous: four Cyperaceae (*Blysmus rufus, Bolboschoenus maritimus, Eleocharis parvula, Schoenoplectus tabernaemontani*), four Juncaceae (*Juncus balticus, Juncus bulbosus, Juncus compressus, Juncus gerardii*), four Poaceae (*Elymus repens, Festuca rubra, Leymus arenarius, Phragmites australis*), and two Typhaceae (*Typha angustifolia, Typha latifolia*). Among dicotyledonous species, Compositae by three species (*Achillea millefolium, Petasites spurius, Tussilago farfara*), one Caryophyllaceae (*Honckenya peploides*), two Convolvulaceae (*Calystegia sepium, Calystegia soldanella*), two Leguminosae (*Lathyrus japonicus, Viccia cracca*), one Primulaceae (*Lysimachia maritima*), one Rosaceae (*Filipendula ulmaria*), one Rubiaceae (*Galium verum*).

Short spacer distances are characteristic for several species with hypogeogenous rhizomes, such as *Schoenoplectus tabarnaemontani*, with characteristic phalanx growth strategy. In contrast, *Typha* spp. and *Phragmites australis* are characterized by long spacing distances, showing a typical guerilla growth strategy.

### 4.2. Achillea millefolium

The presence of *Achillea millefolium* in different coastal habitats has been documented (sand dunes, coastal meadow, salt marsh) [20,30,131,132], and the existence of different ecological races has been proposed [131]. Currently, *A. millefolium* has been recognized as aggregate species [133]. The growth of *A. millefolium* plants was not affected by the salinity of irrigation water up to 5.4 dS m^−1^ [134]. Similarly, the growth of seedlings of *A. millefolium* showed no signs of growth inhibition up to 150 mM NaCl [135]. In natural conditions of salt-affected coastal habitats, Na^+^ accumulation potential in leaves of *A. millefolium* was extremely low vs. relatively high accumulation of K^+^ [29]. Clonal spread of *A. millefolium* can be very fast, up to 25 cm per year [3]; therefore, it would be important to compare the effect of salinity on the clonal growth rate of accessions from different saline and non-saline habitats.

### 4.3. Blysmus rufus

In the United Kingdom, *Blysmus rufus* is a component of coastal vegetation associated with relatively low vegetation zones together with *Juncus articulatus, Juncus gerardi, Eleocharis* spp., *Plantago maritima, Triglochin maritima,* etc. [136]. According to the system of European vegetation classification, *B. rufus* is a component of *Juncetea maritimi* characteristic for “perennial maritime meadows and related herb-rich salt-marshes” [137]. In the Baltic Sea region, *B. rufus* is characteristic component of coastal meadows and the species seems to be competitive only in saline conditions [138]. Obligate the halophyte character of *B. rufus* has been suggested, but prolonged exposure to highly saline conditions evidently resulted in exclusion of the species from typical saltmarsh vegetation [139]. *B. rufus* plants have relatively moderate potential of clonal spread [3]; however, it has not been specifically assessed in field conditions or experimentally. In addition, no information is available on salinity tolerance and ion accumulation potential from studies in controlled conditions.

### 4.4. Bolboschoenus maritimus

*Bolboschoenus maritimus* (syn. *Scirpus maritimus*) is a perennial halophytic clonal species with a rather unique life history among rhizomatous plants. Plants have mostly linear rhizomes, and form shoots, roots, and stem tubers at nodes. Tubers act as overwintering structures but can remain in a dormant state for several years if the water level is low or salinity is high [140]. Vegetative spread by rhizomes can be extremely fast [3]. The species can be found emergent in waters with a wide range of salinity intensity (0.162–30.8 g L^−1^) [141]. In Central European inland salt marshes, *B. maritimus* occurs on soils with a maximum salinity of EC_e_ 63.7 dS m^−1^ [30]. A large volume of information from early studies is available on different aspects of clonal growth and responses to salinity in *B. maritimus*. The information available up to that time has been compiled in a publication in 1996 [141], and readers are encouraged to search for relevant information there. Therefore, mostly information that is not included in it will be further covered in the current review.

**Figure 8:**
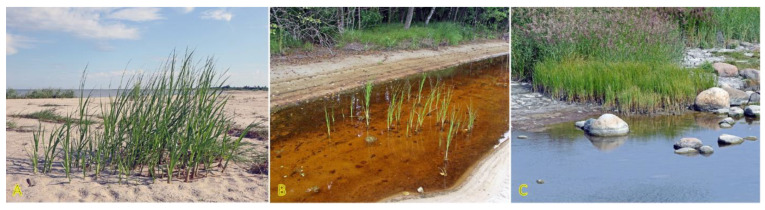
*Bolboschoenus maritimus* on a wet sandy beach in Ainaži, Latvia (**A**), on a wet sandy beach in Melnsils, Latvia (**B**), on a pebble beach on the island of Kihnu, Estonia (**C**).

Traditionally, the species is regarded as a facultative halophyte, but it can tolerate highly alkaline soils [142]. In Sweden *B. maritimus* is characterized as “favoured by moderate salinity, but not restricted to such habitats” [29], and it is included in the eHALOPH database. However, it is sensitive to dry conditions [143]. In wet and flooded coastal habitats, *B. maritimus* plants can be found both as individual genets as well as in large dominant stands (Figure 8).

To understand the possible adaptive advantage of clonality in heterogeneous environments, including salt-affected habitats with fluctuating salinity, analysis of clonal integration in *B. maritimus* is of special importance. Three different types of ramets in *B. maritimus*—with inflorescence-bearing shoots, vegetative shoots, and shoot-less tubers)—are responsible for three main physiological functions: generative, photosynthesis, and storage, respectively [144]. In field conditions, various ramet types were located in particular positions with respect to the elongating rhizome, the main clonal expansion structure: the base position was occupied by flowering ramets, vegetative ramets were situation in first and intermediate positions, but tubers were exclusively located at distal ends of rhizomes. However, this hierarchical structure did not persist in controlled conditions, indicating that specific localization is due to the specificity of environmental conditions. Similar environment-dependent plastic changes can occur with respect to the frequency of particular types of ramets within a genet of *B. maritimus*. Experimental evidence allows the suggestion that the degree of physiological integration in *B. maritimus* is relatively high. Thus, resource translocation between ramets is an important factor for the establishment of new ramets, and even resources stored by dormant interconnected tubers can be used to support sprouting of active tubers [145]. *B. maritimus* can outcompete other co-occurring species only in wet and flooded, but not in dry conditions [146].

*B. maritimus* plants transplanted from salt marsh and exposed to different salinities were able to exhibit stem elongation only at 10 g L^−1^ salinity level, and rapid mortality of individuals started after 2 weeks at 20, 25, and 30 g L^−1^ salinity [147]. Consequently, it appears that actively growing plants are able to tolerate high salinity only for short periods, and habitats with fluctuating salinity regimes are most appropriate for *B. maritimus*. However, even prolonged high-salinity or low-water-level conditions can be survived by means of dormant tubers, showing the importance of clonal plasticity in environmental resilience, and, especially, in salt tolerance. In comparison to other species of the genus, *B. maritimus* has evolutionary adapted to saline habitats at the expense of low phenotypic plasticity with respect to nutrient use efficiency [142]. An inability to increase plant biomass in response to increased nutrient availability by *B. maritimus* is a clear disadvantage of the species in competition in low salinity conditions.

According to the study aimed at assessing ion accumulation characteristics in coastal species from salt-affected habitats, *B. maritimus* was characterized as only moderately Na^+^-accumulating species (4–40 g Na^+^ kg^−1^ DM), regulating tissue electrical conductivity in leaves by the means of changes in K^+^ accumulation [29]. However, no data on ion accumulation can be found from studies performed in controlled conditions.

### 4.5. Calystegia spp.

**Figure 9:**
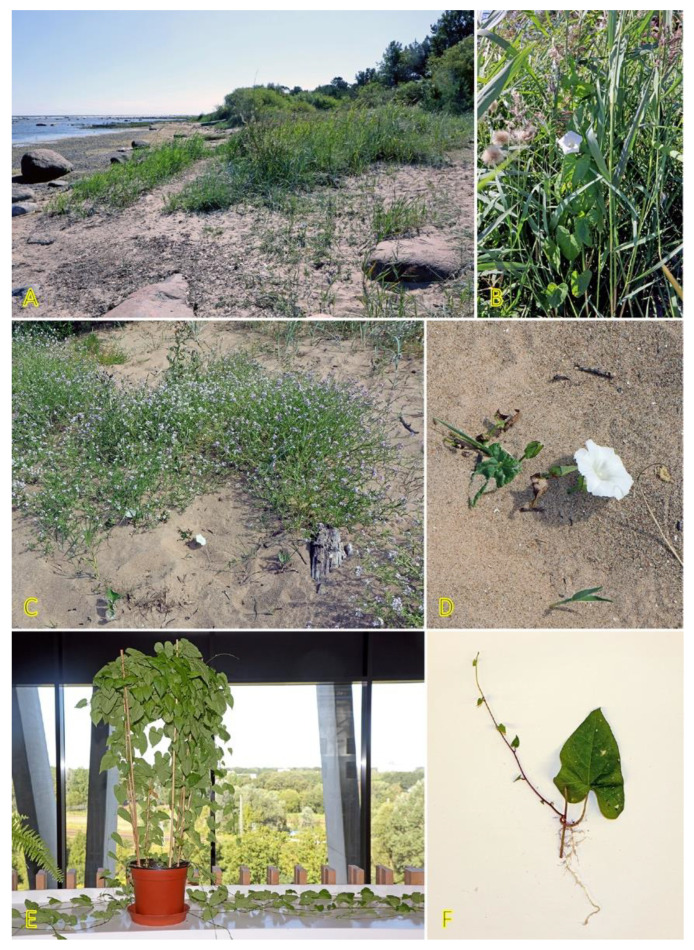
Characteristic coastal sandy beach habitat of *Calsytegia sepium* with *Phragmites australis* in Mērsrags, Latvia (**A**), plants on a beach habitat in Mērsrags, Latvia (**B**), plants on coastal embryonic dunes together with *Cakile maritima* in Salacgrīva, Latvia (**C**) with a detailed view (**D**), formation of plagiotropic rhizome-forming shoots in controlled conditions (**E**), formation of adventitious roots and new shoots at nodes of stem explants with leaf (**F**).

Two clonal species with hypogeogenous rhizomes from the genus *Calystegia*, characteristic of salt-affected coastal habitats, are *Calystegia sepium* and *Calystegia soldanella*. *C. soldanella* is a coastal-specific species of beaches and embryonic dunes [148] and has an importance in dune stabilization [149]; however, *C. sepium* can be found in a wide variety of different inland habitats as well as in salt-adapted coastal plant communities (Figure 9) [28]. Therefore, it was proposed that coastal accessions of *C. sepium* will have higher salinity tolerance in comparison to these from non-saline inland habitats, but it appeared that *C. sepium* has relatively high species-wide salinity tolerance, comparable to that of coastal-specific *C. soldanella* [150]. Only coastal accession of *C. sepium* showed increased accumulation of water in leaves with increasing substrate salinity. The effect of salinity on biomass allocation to clonal growth was an accession-specific trait for *C. sepium*. Plants from both coastal and mesophytic inland accession allocated more biomass to rhizomes with increasing soil salinity, reaching 60 and 20% of the total biomass, respectively, at 260 mmol Na^+^ L^−1^ (6 g Na^+^ L^−1^). This type of response was not evident for *C. sepium* accession from dry grassland, but the response of *C. soldanella* was inconsistent. Interestingly, biomass allocation to rhizomes in *C. sepium* did not depend on nutrient availability in the substrate [151]. In an early study, *C. soldanella* plants were found to be more tolerant to salt spray and salinity inundation than expected from a characteristic, native location with respect to the salinity gradient on the beach [152].

Creeping stems of *C. soldanella* and climbing stems of *C. sepium* do not form adventitious roots in natural conditions, but both can readily form roots and new stems at the nodes of stem explants with leaf (Figure 9F) [150]. In addition, a decrease in the length of the light period for *C. sepium* induces the formation of plagiotropic stems, which grow in the soil and form below-ground overwintering rhizomes (Figure 9E) [153]. Rhizome fragments readily form roots and aerial shoots, and develop new rhizomes [150,154]. In autumn, with an increase of starch content to the maximum, rhizomes enter a dormancy state [155]. For *C. soldanella* individuals, extensive rhizome network development starts only four years after plant establishment from seed, with 3–6 mm thick rhizomes reaching 100 cm soil depth within a diameter of 100–300 cm for 6-year-old individuals [156].

Na^+^ accumulation was restricted in rhizomes of *C. sepium* at high substrate salinity (7–10 g kg^−1^ DM) in comparison to that in leaves (52–73 g kg^−1^) and stems (42–77 g kg^−1^) [150]. Both species accumulated inorganic ions as well as non-ionic osmotica as a means of osmotic adjustment. In natural conditions of salt-affected coastal habitats, *C. sepium* had characteristics of a tight regulator of electrical conductivity level in leaves, with proportional adjustment of Na^+^ and K^+^ concentration [29].

**Figure 10:**
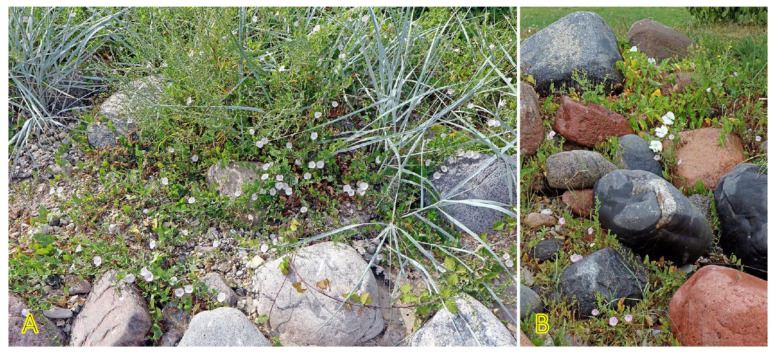
*Convolvulus arvensis* on a stony beach together with *Leymus arenarius* on the island of Kihnu, Estonia (**A**), on a stony beach together with *Calystegia sepium* on the island of Saaremaa, Estonia (**B**).

Another taxonomically related and morphologically similar climbing clonal species, *Convolvulus arvensis*, can be relatively often found in coastal habitats [157], often together with *C. sepium* (Figure 10). The species is not included in the eHALOPH, and its salinity tolerance has been characterized as low [158], but the ecological indicator value with respect to salinity for *C. arvensis* in Sweden has been indicated as 2 out of 5 [27].

### 4.6. Eleocharis spp.

Among the European species of the genus *Eleocharis*, there are three halophytes: *Eleocharis palustris, Eleocharis parvula*, and *Eleocharis uniglumis*, included in the eHALOPH database. Presumably the least salt-tolerant species is *E. palustris* (salinity tolerance level 2 out of 5), followed by *E. uniglumis* (salinity tolerance level 3), and *E. parvula* (salinity tolerance level 4) [27]. All of them have clonal growth organs, dominant hypogeogenous rhizomes, with an ability to form also epigeogenous rhizomes [3]. *E. parvula* is the only species out of the three having the third type of clonal growth organ, stem tubers. Similar to *Bolboschoenus maritimus*, tubers of *E. parvula* act as overwintering and reserve-bearing organs.

*E. parvula* is characteristically found in salt marshes, mudflats, brackish wet meadows, and similar habitats where it forms dense short stands, often in flooded conditions [159]. It can be present together with visually similar species, *Blysmus rufus, Juncus gerardi*, *Juncus bulbosus* etc. [160]. No studies so far have focused on the ecophysiology of *E. parvula* or its salt tolerance.

### 4.7. Elymus repens

*Elymus repens* (syn. *Agropyron repens, Elytrigia repens*), a rhizomatous grass species with a guerilla clonal growth strategy, has been included in the eHALOPH database and has been characterized as moderately salinity tolerant in Sweden [27]. Communities with a high abundance of *E. repens* are common for typical upper salt marshes [161]. In Central European inland salt marshes, the species occurs with a relatively high frequency (28%) and can be found on soils with a maximum salinity of EC_e_ 45.5 dS m^−1^ [30]. In the Southern and Eastern Baltic, *E. repens* has been identified as a community-forming species on soils with relatively high Na^+^ concentration (1.4 g L^−1^) [28]. *E. repens* has been found also in shore meadows along the Bothnian Sea [162]. In natural conditions of Mediterranean salt marsh, the frequency of *E. repens* increases with decreasing salinity [163]. No studies so far experimentally assessed the salinity tolerance of *E. repens*.

The extensive branched rhizome system of *E. repens* allows for fast colonization of land by forming large clonal patches [164]. Therefore, the species has been characterized as a “below-ground integrator” [14]. In disturbed habitats, rhizome fragments act as propagules for regrowth, using stored fructan reserves [165]. In heterogeneous environments, *E. repens* plants use an exploitive strategy by placing rhizomes in resource-rich patches [166]. In other terms, the species use a clonal escape strategy to move out from unfavorable soil patches by producing long rhizomes [167]. It was shown that clonal integration is important for the early phases of beach colonization by *E. repens* [168]. In the context of salinity tolerance, it would need to be clarified if *E. repens* plants derive adaptive advantages from clonal integration under heterogeneous salinity conditions.

Morphologically similar rhizomatous grass species *Spartina alterniflora* showed identical negative responses on vegetative growth and sexual reproduction by salinity, and plants clearly benefited from clonal integration in conditions of severe salinity [169]. Physiological integration has been shown to be an important feature of two other rhizomatous salt marsh grasses, *Spartina patens* and *Distichlis spicata* [170]. In particular, in hypersaline conditions that inhibit seed germination, clonal grasses with parent ramets located in low-salinity patches can place daughter ramets in patches with high salinity, supporting their growth by sharing water and carbohydrate reserves.

### 4.8. Festuca rubra

*Festuca rubra* is a rhizomatous species that has been associated with a variety of habitats, including the ones affected by salinity, and its salinity tolerance has been evaluated as moderate [27]. In addition, the species has been included in the eHALOPH database. Given the wide range of environmental conditions in habitats with *F. rubra*, the existence of different ecotypes can be proposed. In the Netherlands, three subspecies of *F. rubra* have been recognized depending on the location of their habitat [171]. In Baltic coastal wetlands, *F. rubra* is one of the most abundant species in lower-shore and upper-shore communities [19]. In Central European inland salt marshes, the species occurs with a relatively high frequency (17%) and can be found on soils with a maximum salinity of EC_e_ 25.7 dS m^−1^ [30]. Adaptive genetic differentiation of *F. rubra* indeed has been demonstrated within populations along crossed gradients of moisture and temperature [172].

Different ecotypes of *F. rubra* from salt marshes, sand dunes, and inland sites were compared in conditions of hydroponics with respect to their salinity tolerance [173]. As a result, the ecotype from salt marsh (*F. rubra* subsp. *litoralis*) was characterized as salt-tolerant (being able to tolerate 300 mM NaCl), the ecotype from the sand dune (*F. rubra* subsp. *arenaria*) as medium tolerant (being able to tolerate 150 mM NaCl but with almost completely inhibited growth), and the inland ecotype (*F. rubra* subsp. *rubra*) as salt-intolerant (as unable to sustain in 150 mM NaCl). In another study, the salt marsh ecotype of *F. rubra* showed moderate tolerance to salinity, with significant growth inhibition already at 50 mM NaCl concentration, and root growth was more affected [174].

Several studies have assessed aspects of clonal architecture and genet demography of *F. rubra* in mountain habitats [175,176], but no information is available on the effects of salinity on clonal growth characteristics of the species. Analysis of this relationship in relation to genetic differentiation would be particularly useful.

### 4.9. Filipendula ulmaria

*Filipendula ulmaria* is a characteristic plant species of the Baltic seashore meadow vegetation [162,177]. The species is not included in the eHALOPH database, and it has been characterized as “moderately salt-tolerant, but preferring non-saline conditions” [27]. In the United Kingdom, *F. ulmaria* can be found together with *Iris pseudacorus* on the upper edges of boulder beaches [136]. Clonal architecture of *F. ulmaria* as an integrative part of individual growth has been studied experimentally, and it was concluded that relatively slow but long-term expansion of genets is followed by fragmentation facilitated by accumulation of necrotic remains of both shoots and rhizome [178]. Fast growth following genet disintegration allows the species to quickly colonize new areas. There is no information available on experimentally obtained data on the salinity tolerance of *F. ulmaria*. In conditions of the saline habitats of the Baltic Sea, the leaves of *F. ulmaria* accumulated very low concentrations of Na^+^ [29]. There is an extremely large practical interest in this species due to the potential use of the plant in pharmacology [179].

### 4.10. Galium verum

**Figure 11:**
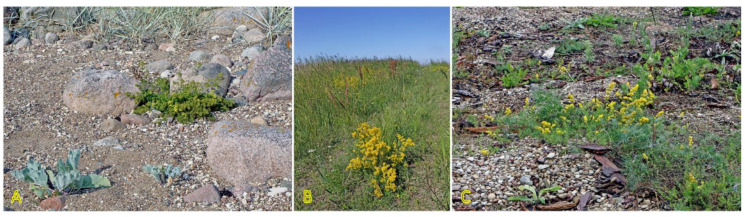
*Galium verum* plants together with *Crambe maritima* and *Leymus arenarius* on a gravel beach on the island of Kihnu, Estonia (**A**), on a coastal sandy meadow in Kuiviži, Latvia (**B**), on a gravel beach on the island of Saaremaa, Estonia (**C**).

*Galium verum* is relatively often found in coastal habitats, as relatively dry parts of coastal grasslands, fixed dunes, seashore meadows, as well as stable gravel and pebble beaches (Figure 11). The species has been characterized as only moderately salinity tolerant [27]. However, no salinity tolerance of *G. verum* has been experimentally assessed, but the Na^+^ accumulation potential of plants from coastal habitats was characterized as low [29]. *G. verum* is a clonal species with an ability for relatively fast vegetative spread [3]. The majority of ecologically oriented research performed on *Galium verum* has been with respect to dune grasslands [180,181,182,183]. Effects of both resource heterogeneity and nutrient availability have been studied, and significant effects were shown with respect to the abundance of *G. verum* plants, but no aspects of clonal growth have been analyzed.

### 4.11. Honckenya peploides

**Figure 12:**
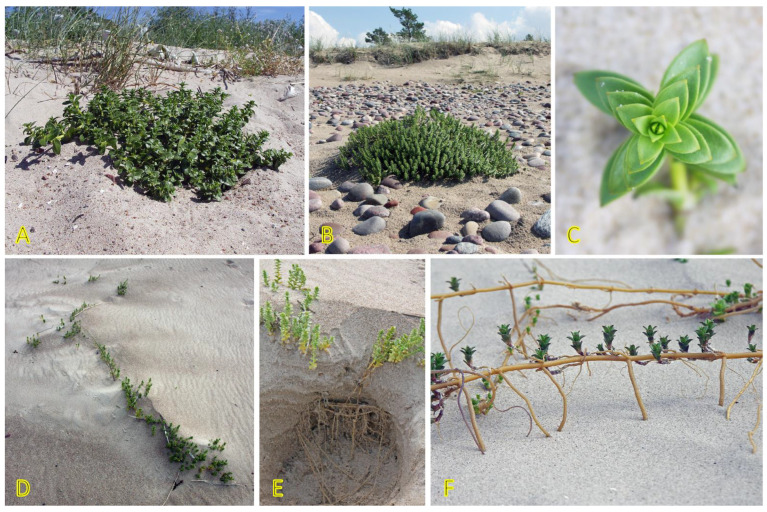
*Honckenya peploides* plants on undisturbed dunes in Kuiviži, Latvia (**A**), on a gravel–pebble beach in Užava, Latvia (**B**), a symmetric shoot (**C**), foraging genet on embryonic dunes in Carnikava, Latvia (**D**), excavated rhizomes in Carnikava, Latvia (**E**), genet on an eroded sandy beach in Buļļusala, Rīga, Latvia (**F**).

*Honckenya peploides,* often found on embryonic dunes, is one of a few dune-building dicotyledonous species [184]. From a biological point of view, the species is extremely interesting, both with respect to peculiarities of the reproductive system as well as clonal behavior. Individuals of *H. peploides* are either female never producing pollen or hermaphroditic producing mainly pollen but also a small number of seeds [185]. While the species spread by means of rapidly expanding rhizomes, it seems that the specific clonal growth pattern for each genet depends on particular environmental conditions, mainly the stability of the sand level. In relatively stable conditions, distinct individuals with irregularly arranged leaves of varying size are formed (Figure 12A), but after being disturbed, most likely by burying in the sand, plants develop shoots with strongly regularly arranged leaves gradually decreasing in size towards the top (Figure 12B,C). In some cases, a pronounced foraging response can be observed when the arrangement of the shoots in the lines (Figure 12D) is related to the location of rhizomes in the upper layer of the soil (Figure 12E). The plants also show remarkable resistance to sand erosion, where thick rhizomes form a support system to maintain the shoots in an upright position (Figure 12F). Thus, extreme plasticity of clonal growth form variation in *H. peploides* can be seen, evidently leading to adaptive resilience of the species.

The species has been listed in the eHALOPH database, and its salinity tolerance in Sweden is evaluated as being “competitive only under moderate-high salinity” [27]. Seedlings of *H. peploides* collected on subarctic coastal dunes showed good general salinity tolerance, as 40% of seedlings were able to survive at 10 g L^−1^ sea salt, but only a few seedlings (13%) survived at 20 g L^−1^ salinity [186]. Growth of both shoots and roots was inhibited already at 5 g L^−1^, and additional salt spray further inhibited growth. In a study where spray with sea water was performed separately on female and male individuals, it was shown that it had a positive effect on root and shoot growth only on the background of nutrient addition-stimulated growth [187]. Using stem apical and nodal explants for propagation in tissue culture, it was evident that root growth was inhibited already at 25 mM NaCl in cultivation medium, but shoot growth was negatively affected only at 75 mM NaCl [188].

It is very surprising that no study so far has addressed the problem of salinity tolerance in *H. peploides* in necessary detail, assessing the effect of different types of salinity on ion accumulation, osmotic protection, as well as plant water status. Studies of ion accumulation suggest that in native habitat *H. peploides* plants accumulate variable Na^+^ concentrations in leaves, ranging from low to moderate, together with low K^+^ concentration, on a background of moderate-to-high water content, leading to relatively low electrical conductivity on a tissue water basis [29].

### 4.12. Juncus spp.

Among several species of the genus *Juncus* found in coastal habitats, only *Juncus balticus* and *Juncus gerardii* have high salinity tolerance (indicator value 4 out of 5) [27], and they are listed in the eHALOPH database. In addition, *Juncus compressus* has an indicator value for salinity tolerance of 3 out of 5. All three species are characterized by similar clonal growth systems (hypogeogenous rhizomes) and relatively comparable potential of clonal spread [3]. Another species of the genus, seldom found in coastal habitats, *Juncus bulbosus*, has both epigeogenous and hypogeogenous rhizomes as well as stolons, and is characterized as sensitive to salinity.

**Figure 13:**
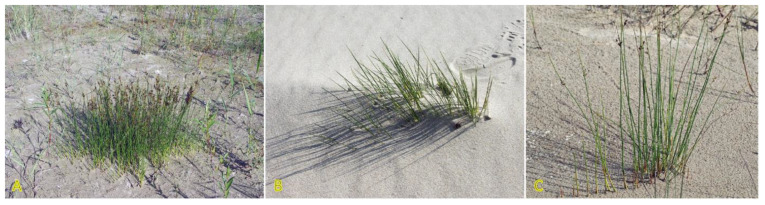
Genets of *Juncus balticus* on a wet beach in Lielupe, Jūrmala, Latvia (**A**), on embryonic dunes in Ovīši, Latvia (**B**), on a wet beach in Vaide, Latvia (**C**).

*J. balticus* is commonly found in wet beach habitats (Figure 13), dune slacks, and various permanently wet coastal habitats [189]. Rhizomes with long internodes give rise to scattered stems, but a characteristic type of branching makes genets easily distinguishable from other rush species (Figure 13A). However, particular clonal growth characteristics have not been studied. Similarly, so far, no studies have experimentally assessed the salinity tolerance of *J. balticus*, but the Na^+^ accumulation potential of the species in natural conditions seems to be extremely low [29].

*Juncus gerardii* is a characteristic upper salt marsh species, but can be found in different wet habitats with permanent perennial vegetation and even as a pioneer species [190]. In Baltic coastal wetlands, *J. gerardii* is one of the most abundant species in lower-shore and upper-shore communities [19]. Annually formed ramets within a genet with a phalanx clonal growth strategy have a very high degree of physiological integration [190]. Clonal architecture of *J. gerardii* had not been significantly affected by defoliation [191], and species abundance was even slightly stimulated by increased grazing intensity [192]. Moreover, the abundance of *J. gerardii* decreased with decreasing salinity [19].

In controlled conditions, treatment with 340 mM NaCl resulted in 60% inhibition of growth in terms of biomass accumulation, and combined treatment of salinity with waterlogging resulted in 77% biomass reduction [120]. The more pronounced negative effect of waterlogging was associated with an increase in shoot Na^+^ concentration from 41.3 g kg^−1^ in saline conditions to 154 g kg^−1^ in waterlogged conditions. However, no significant changes in K^+^ concentration occurred under salinity or combined treatments. In another study, the growth of *J. gerardii* was not affected by 50% seawater treatment, but biomass accumulation, as well as shoot and rhizome elongation inhibited by flooding [46]. The negative effect of flooding was also found in studies on field conditions [193].

### 4.13. Lathyrus japonicus

**Figure 14:**
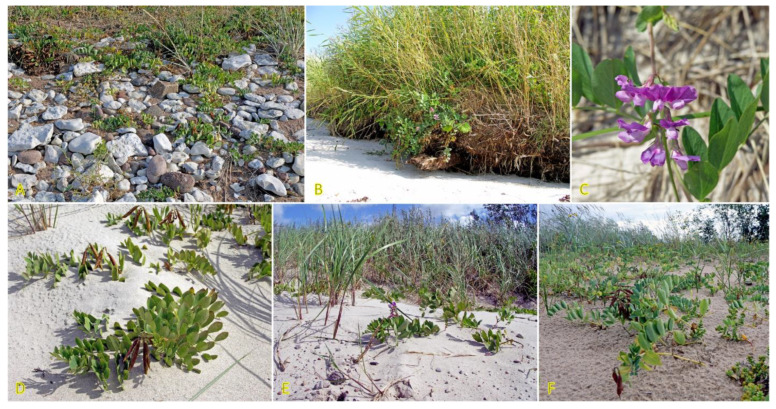
*Lathyrus japonicus* plants on a stony beach on the island of Hiiumaa, Estonia (**A**), on the edge of a marshy meadow together with *Phragmites australis* in Jūrmalciems, Latvia (**B**), detail of flowering individual in Jūrmalciems, Latvia (**C**), on embryonic dunes in Pape, Latvia (**D**), on white dunes in Užava, Latvia (**E**), on white dunes in Carnikava, Latvia (**F**).

*Lathyrus japonicus* is just one of a few clonal legume species included in the eHALOPH database. The species is globally distributed on sand and shingle beaches or dunes across the temperate zone (Figure 14) [194]. Seeds are very tolerant to floating in seawater while maintaining viability for up to five years [195]. Sporadic appearance of *L. japonicus* plants has been associated with the establishment of drift seeds stranded on the shore [196]. Scarification of the seed coat by abrasive action against coarse substrate is the only way to make them permeable to water [197]. Local clonal distribution by hypogeogenous rhizomes is relatively fast and can reach 29 cm per year [3]. Maximum ramet density of *L. japonicus* was found in foredunes 15 m from the embryonic dunes, linearly decreasing inland [132].

Based on the appearance of *L. japonicus* in relatively salt-affected habitats, in the Baltic region, it has a salinity tolerance of 4 out of 5, as being “competitive only under moderate-high salinity” [27]. Similarly, the species has been listed among halophytes in China [198]. However, not much experimental evidence so far are available to prove the salinity tolerance of *L. japonicus*. In one comparative study, *L. japonicus* plants were indicated to be more tolerant to salt spray and salinity inundation than expected from a characteristically native location with respect to the salinity gradient on the beach [152]. However, no particular results from actual measurements were provided. In natural conditions, Na^+^ accumulation potential in leaves was very low [29].

### 4.14. Leymus spp.

Among the typical dune-building grasses, several species of the genus *Leymus*, including *Leymus arenarius, Leymus chinensis* and *Leymus mollis*, are listed in the eHALOPH database. These species have different distribution ranges, as *L. arenarius* is native to northern European sand dunes, *Leymus chinensis* is native to Asia, but *Leymus mollis* is native to Asia and North America.

The existence of a genotype-specific salinity tolerance has been shown for *L. arenarius* when plants from a coastal population were able to maintain higher root dry mass, number of tillers, as well as leaf area with increasing salinity in comparison to the plants from inland populations [199]. No detailed studies have been performed on the possible effect of salinity on clonal growth characteristics in *L. arenarius*; however, this type of response had been assessed for *L. chinensis*, a species showing extreme tolerance to grazing and saline-alkali conditions [200]. It seems that the species efficiently use clonal integration in order to compensate for the salinity-induced decrease in ramet density with an increase in the biomass of individual ramets. Also, defoliation of *L. chinensis* plants cultivated in low salinity conditions promoted rhizome elongation and establishment of new ramets, resulting in an expansion into saline-alkali soil patches [201]. Moreover, in a homogeneous environment, leaf clipping resulted in the inhibition of biomass accumulation and rhizome expansion, but such an effect was insignificant for plants grown in soil with saline-alkaline patches [202].

As a positive response of dune-building grass species towards sand accretion, is a critical feature for the resilience of these species in dune habitats and species-specific responses to sand burial associated with differences in clonal growth characteristics, evident [203], it would be important to understand combined effects of salinity vs sand accretion on the physiological status and growth aspects of *L. arenarius*. In Central European inland salt marshes, *L. maritima* is relatively frequent (21%) and appears on soils with a maximum salinity of EC_e_ 118.0 dS m^−1^ [30].

### 4.15. Lysimachia maritima

**Figure 15:**
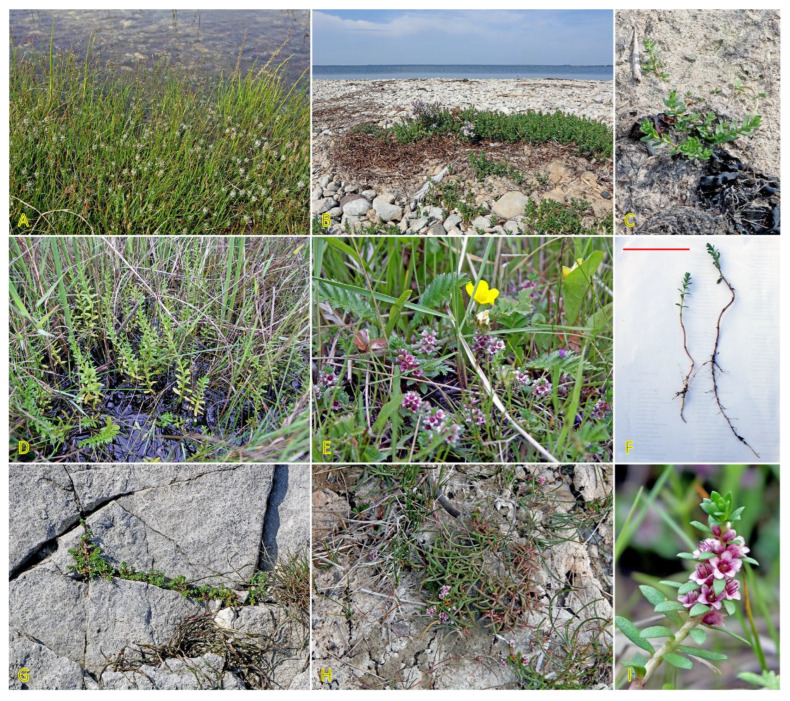
*Lysimachia maritima* plants together with *Juncus* sp. and *Triglochin maritima* on a wet coastal meadow on the island of Saaremaa, Estonia (**A**), on a shingle beach on the island of Hiiumaa, Estonia (**B**), on a wet sandy beach in Ainaži, Latvia (**C**), flooded coastal meadow in Liepāja, Latvia (**D**), flowering individuals together with *Potentilla anserina* on a wet coastal meadow in Mērsrags, Latvia (**E**), excavated individuals (**F**), on a rocky beach on the island of Kihnu, Estonia (**G**), on a gravel beach together with *Spergularia marina* on the island of Saaremaa, Estonia (**H**), flowering shoot (**I**).

*Lysimachia maritima* (syn. *Glaux maritima*) is a species exclusively found in coastal habitats, where it can occupy a wide range of niches on salt-affected soils with various environmental conditions (Figure 15) [27]. In the Baltic coastal wetlands, *L. maritima* is the most abundant species in the open pioneer community [19]. Therefore, from a point of ecological indication, it is characterized as “competitive only under moderate–high salinity” [27] and included in the eHALOPH database. In Central European inland salt marshes, *L. maritima* is relatively frequent (21%) and appears on soils with a maximum salinity of EC_e_ 118.0 dS m^−1^ [30].

*L. maritima* plants have specific life stage characteristics, as particular overwintering structures (hibernacles) are formed on rhizomes; only two or three per plant [204]. Hibernacles are in a dormant state in autumn and require a cold period of at least several weeks to get into a state capable of growth [205]. Rhizomes are relatively short-lived and serve mostly as a means for placing hibernacles at some distance from the mother plant [206]. Consequently, clonal growth in *L. maritima* serves exclusively for vegetative propagation with no long-term connection between the formed ramets and no opportunity for physiological integration. Rhizome elongation was stimulated in conditions of low competition, high nutrient level, or high light intensity, but plants remained stationary in dense vegetation conditions [207]. Vegetation grazing and flooding had similar effects on population dynamics. Ecotypic differentiation of *L. maritima* plants along an environmental gradient was proposed because of variation in response patterns to different light intensities and inundation in a common garden experiment [208]. However, as vegetatively propagated plant material was used in this study, there is a high probability that the observed differences were caused by epigenetic changes.

Growth stimulation of *L. maritima* plants by moderate salinity has been indicated both in sand culture experiments in a greenhouse [209] as well as in conditions of tissue culture [210]. Even 300 mM NaCl had no negative effect on plant growth, but inundation resulted in growth inhibition irrespective of salinity [209]. However, in a field study, it was shown that *L. maritima* plants exhibiting flooding with seawater pulse responded to a decline in the photochemical efficiency of photosystem II and a decrease in frequency and intensity of mycorrhizal symbiosis [211]. Another typical factor in sandy coastal habitats, sand accretion, tended to increase shoot and root biomass (when the accretion level was 5 cm), and increased both the number and mass of hibernacles; however, these parameters were diminished by more intense sand accretion by 10 cm [209].

The salt accumulation potential of *L. maritima* plants can be evaluated as above average but not exceptional, based on studies both in natural and controlled conditions. In leaves of plants from coastal habitats, the median concentration value for Na^+^ was 20 g kg^−1^ DM [29]. However, during a seasonal pulse of soil salinity, Na^+^ concentration in leaves of *L. maritima* shortly reached 100 g kg^−1^, with a decline to a base level at 18 g kg^−1^ further in the season [212]. In cultivated plants subjected to 300 mM NaCl, shoots and roots accumulated 28.0 and 5.6 g Na^+^ kg^−1^ DM, respectively, but flooding resulted in an increase up to 43.5 and 45.0 g kg^−1^ DM, respectively [209]. Relatively moderate ion accumulation capacity of *L. maritima* might be related to the fact that about 20% of all absorbed sodium is secreted to salt glands [213]. K^+^ concentration in shoots decreased by increasing salinity [204], confirming that electrolyte level in shoots of *L. maritima* is controlled mainly by changes in Na^+^ concentration [29].

### 4.16. Phargmites australis

**Figure 16:**
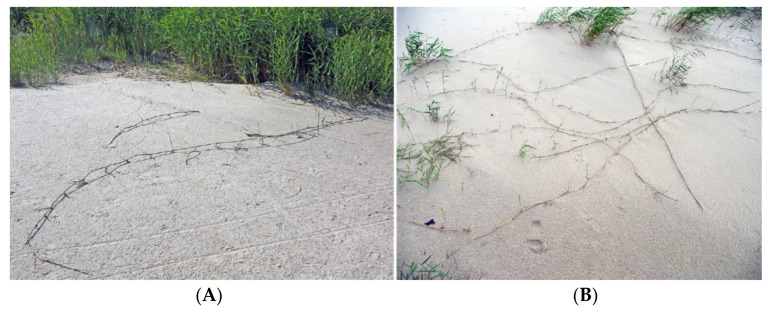
Foraging clonal structures of *Phragmites australis* on a wet beach in Ainaži, Latvia (**A**) and in Lielupe, Jūrmala, Latvia (**B**).

*Phragmites australis* is a clonal species with one of the highest potentials for clonal spread [3]. Foraging behavior of *P. australis* by stolons can be frequently observed in conditions of open wet sandy beaches (Figure 16). The species has been relatively well studied for the effects of salinity and other factors on growth and distribution in both natural and controlled conditions. This is largely due to the fact that it is considered invasive or aggressively dominant in several regions of the world. *P. australis* is extremely abundant in Central European inland salt marshes (frequency 33%) and occurs on soils up to EC_e_ 118.0 dS m^−1^ [30].

In greenhouse conditions, increasing salinity (15 and 30 g L^−1^) progressively decreased both the culm height and density of *P. australis* plants, as well as above-ground biomass and amount of carbohydrate reserves in rhizomes [214]. However, 10 g L^−1^ salinity had no negative effect. It is also important that salinity tolerance differed between plants produced from seeds and those grown from rhizomes [215]. Thus, while no mortality was evident for both types of plants in hydroponics at 15 g L^−1^ salinity, the survival rate at 22.5 g L^−1^ salinity was 75 and 12%, for rhizome-grown plants and seedlings, respectively. No plants survived at 35 and 50 g L^−1^ salinity. For seedlings, increasing salinity resulted in decreased growth rate, but the growth of rhizome-derived plants was optimum at 5 g L^−1^ salinity. Moreover, high soil nutrient heterogeneity can increase the negative effect of salinity on plant growth, as found in the study under controlled conditions [216]. In another study, 1.5 g L^−1^ NaCl did not result in growth inhibition, but all plants died at 35 g L^−1^ salinity [217].

Thus, it seems that the salinity tolerance in *P. australis* is a genotype-dependent trait. Salinity tolerance of 15 various clones of different geographic origins was compared [218]. All clones survived 410 mM NaCl for 14 days, but selective mortality was observed at 547 mM. Only three clones were able to survive at 1230 mM NaCl. The degree of growth inhibition also depended on the genotype and generally correlated with the survival ability of the given genotype. In another study, salt tolerance was compared for *P. australis* plants from nine populations from coastal Mediterranean salt marshes [219]. A growth decrease of 50% was evident already at 7.5 g L^−1^ NaCl for 25 days, but population-specific individual mortality occurred at 15 and 20 g L^−1^ NaCl. Most importantly, no variation in morphological response patterns to salinity was related to the environmental conditions in the natural populations.

Adaptations to salinity were studied in natural conditions of river delta wetland ecosystems, and it was found that increasing salinity was more important for morphological and physiological variability in comparison to the effects of soil water content [220]. In particular, plant height, leaf area, and stem diameter decreased but leaf water content increased with increasing salinity. It has been established under natural conditions that the invasion of the species in salt-affected marshes depends on local changes in conditions separated in time, the so-called opportunity windows. Thus, successful establishment by seed or rhizome fragments is possible when salinity drops below 10 g L^−1^, sulfide concentration is less than 0.1 mM, but flooding frequency is below 10% [221,222]. However, established stands can easily tolerate 45 g L^−1^ salinity, 1.75 mM sulfide, and up to 100% flooding frequency [222]. The negative effect of both NaCl and sulfide was related to the reduced ability of *P. australis* plants for the uptake of mineral elements, especially nitrogen [223]. However, in freshwater marshes, saltwater intrusion might have a promoting influence on the invasion of *P. australis* due to the indirect effect of salinity through the alteration of soil microbial composition [224].

Na^+^ accumulation potential in roots of *P. australis* in controlled conditions was 23–35 g kg^−1^, but only 9.2–17.3 g kg^−1^ in stems and 4.6–6.9 g kg^−1^ in leaves [225]. Besides, the maximum accumulation in stems and leaves was already at 100 mM NaCl. Similar Na^+^ content values were found in leaves of *P. australis* plants in salt-affected coastal habitats, and species seemed to control leaf EC by changes in K^+^ concentration [29]. Certain genotype-dependent differences in the Na^+^ accumulation pattern within a plant were evident for the most salt-tolerant clones at high salinity [218]. In particular, 27.6–43.7 g Na^+^ kg^−1^ DM was accumulated in roots, 9.2–27.6 g kg^−1^ in stems, 5.8–28.8 g kg^−1^ in leaves, and only 9.2–15.0 g kg^−1^ in rhizomes.

Nutrient allocation strategies were studied for *P. australis* plants grown in natural conditions in different habitats: wetland, salt marsh, and desert [226]. Soil water content decreased, but soil salinity increased in the direction from wetland to desert and the occurrence of plants decreased in this direction. The concentration of C, N, and P increased in shoots and decreased in rhizomes of plants in the direction from wetland to desert, suggesting that *P. australis* plants allocate more resources to clonal growth with an increase of intensity of adverse environmental factors. In general, a relatively high degree of clonal integration seems to be an important characteristic of *P. australis* for establishment and expansion in marsh habitats [227].

### 4.17. Schoenoplectus tabarnaemontani

*Schoenoplectus tabernaemontani* is characterized by high clonal spread potential [3]. Resource allocation to daughter ramets during vegetative spread clearly depends on the rhizome biomass of parent ramets but not on their stem biomass, showing the importance of stored resources in rhizomes [228]. Aspects of clonal growth of *S. tabernaemontani* plants have been studied with respect to mineral nutrient heterogeneity (both vertical and horizontal) and flooding depth [229]. In homogeneous nutrient conditions, with an increase in flooding intensity, plant growth was stimulated, and more biomass was allocated to shoot growth, but the number of ramets decreased. However, in heterogeneous nutrient conditions, the effect of increased flooding depth on growth tended to be negative, and the number of ramets still decreased. In another study, *S. tabernaemontani* plants were exposed to the influence of mineral nutrient heterogeneity, and this resulted in a decrease in total biomass, mostly at the expense of belowground biomass [230]. However, the species showed no growth responses to increased nutrient concentration in conditions of the mesocosm study, and it even decreased with an increased nutrient concentration in natural conditions, when present together with *Typha latifolia* [231].

**Figure 17:**
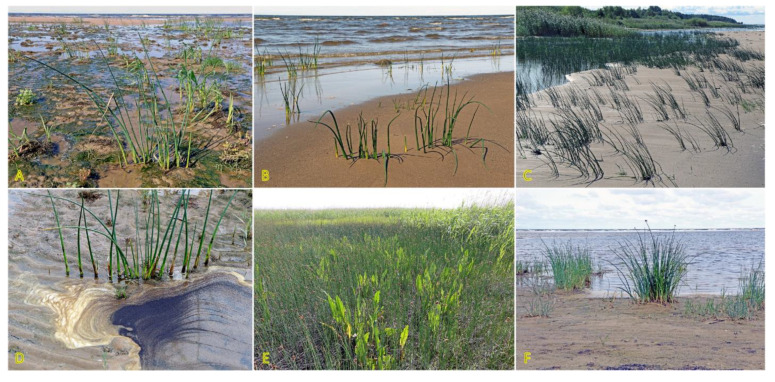
*Schoenoplectus tabernaemontani* on a permanently wet sandy beach with developing perennial vegetation in Salacgrīva, Latvia (**A**), on a wet sandy beach in Ainaži, Latvia (**B**), on a flooding sandy beach on the edge of the permanent lagoon in Ainaži, Latvia (**C**), on a permanent puddle in Ainaži, Latvia (**D**), on salt marsh together with *Rumex hydrolapathum* and *Bolboschoenus maritimus* in Mērsrags, Latvia (**E**), on a wet sandy beach in Lielupe, Jūrmala, Latvia (**F**).

*S. tabernaemontani* is included in the eHALOPH database and it is characterized as “competitive only under moderate–high salinity” (indicator value 4 out of 5) [27]. In coastal habitats of the Baltic Sea, *S. tabernaemontani* plants can be found not only in permanent and temporary lagoons, salt marshes, and wet subhalophilous meadows [28,177], but also on permanently wet sandy beaches (Figure 17). *S. tabernaemontani* plants primarily occur at salinities < 5 g L^−1^, but can withstand pulses of 10 g L^−1^ salinity [232]. In a model study, plant height was not significantly affected by inundation with brackish water (10 g L^−1^, EC 15.36 mS cm^−1^) either permanently or as salinity pulses, in comparison to freshwater, but root/shoot ratio tended to decrease [233]. In natural conditions, *S. tabernaemontani* plants showed only moderate Na^+^ accumulation potential, but were also able to accumulate relatively high amounts of K^+^, in general showing high tissue electrolyte concentration [29].

Being typically emergent macrophyte species, *S. tabernaemontani* is relatively tolerant to moderate inundation, but is susceptible to deep water (>120 cm) or long periods of flooding [234]. From the point of view of ecosystem services, *S. tabernaemontani* is important for shoreline protection, erosion control as a stabilizing factor, as a habitat for other species, as well as for providing aeration of sediment and water [235]. In addition, the species has been used in different practical applications of phytoremediation, such as for desalination in bioreactor systems for greenhouse effluent treatment [236].

### 4.18. Tussilago farfara

**Figure 18:**
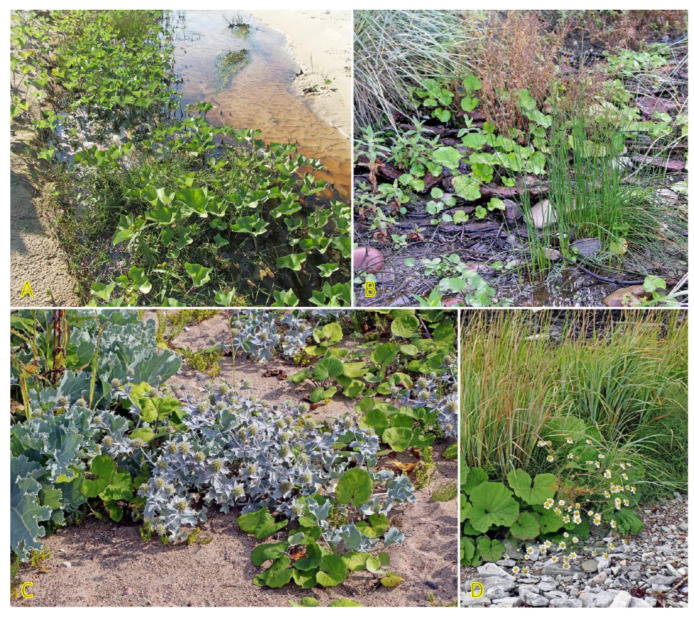
*Tussilago farfara* in the mouth of a stream on a sandy beach in Mazirbe, Latvia (**A**), on a spring-affected sandy beach together with *Juncus balticus* in Liepene, Latvia (**B**), on a sandy beach together with *Eryngium maritimum* and *Crambe maritima* on the island of Kihnu, Estonia (**C**), on a pebble beach with perennial vegetation together with *Tripleurospermum maritimum* on the island of Hiiumaa, Estonia (**D**).

*Tussilago farfara* is a typical pioneer species, usually colonizing various open and ruderal habitats by long-distance wind dispersal of seeds and further expanding locally by means of long branching rhizomes, resulting in guerilla-type clonal growth [237]. Generative and vegetative stages are separated, as flowering stems without leaves are formed in early spring, followed by growth of leaves and clonal parts later in the season [238]. Rhizomes contain carbohydrate resources, water, and minerals to sustain flowering during the leafless period as well as growth of leaves and new clonal structures [239]. Spacer lengths can reach up to 90 cm [240]. Disintegration of rhizome fragments occurs readily, further promoting clonal expansion [240]. While not listed in the eHALOPH database, *T. farfara* is often found in sea-affected coastal habitats (Figure 18). However, it is only characterized as “moderately salt tolerant, but preferring non-saline conditions” [27].

The potential for vegetative regrowth after disturbance that causes rhizome fragmentation is quite extraordinary. Thus, 6 cm long rhizome fragments can emerge from 42 cm depth and exhibit normal development [241]. As a result, even complete destruction of vegetation is favorable for the renewal of *T. farfara* population [238].

Environmental conditions affect generative vs vegetative reproduction when plants in less optimal conditions allocate more resources toward clonal growth [242]. In particular, planting density and soil fertility effects on generative vs vegetative reproduction have been studied for *T. farfara* [239]. In soil with a low level of available mineral nutrients, plants tended to allocate proportionally more biomass towards vegetative reproduction, without reducing allocation to sexual reproduction. However, an increase in plant density from medium to high significantly reduced biomass allocation to clonal growth. Resource allocation to rhizomes decreased also with decreasing light intensity [243].

There is no information available on the experimentally assessed salinity tolerance of *T. farfara*. However, high tolerance to alkaline conditions can be expected [243]. In conditions of salt-affected coastal habitats, the species accumulate extremely low concentrations of Na^+^ in leaves, regulating tissue EC by changes in K^+^ concentration [29]. Identification of salt-tolerant ecotypes of *T. farfara* could be important from both theoretical and practical points of view. It would be essential to determine whether increased salinity causes enhanced clonal growth, at least, in relative terms. Regarding the practical importance of *T. farfara*, on the one hand, it is a noxious weed and on the other, a valuable medicinal plant [244]. Besides, a possibility to use *T. farfara* in the reclamation of degraded lands has been successfully tested [245].

Taxonomically related species *Petasites spurius* [237] with similar life form characteristics can be often found on coastal beaches and dunes [29], and it is also characterized as moderately salt tolerant [27]. The species has received attention in the context of its pharmacological use [246].

### 4.19. Typha spp.

**Figure 19:**
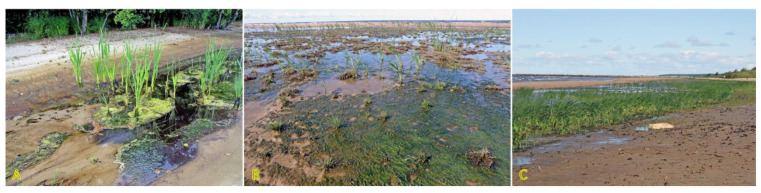
*Typha* sp. on a wet sandy beach in Melnsils, Latvia (**A**), on a wet sandy beach with developing perennial vegetation in Ainaži, Latvia (**B**), on a permanent coastal lagoon on a wet sandy beach in Salacgrīva, Latvia (**C**).

Two species of the genus *Typha* with relatively similar clonal growth characteristics, distributed throughout the world in wetland habitats, *Typha angustifolia* and *Typha latifolia*, are commonly found in different coastal habitats (Figure 19). Both are suggested to represent halophytes, and are included in the eHALOPH database, but their salinity tolerance has been characterized as moderate [27]. *T. angustifolia* is characteristic of sites with deeper waters and plants of this species have larger salinity tolerance, but *T. latifolia* is found on sites with more shallow waters and possesses lower salt tolerance [247,248]. However, *T. angustifolia* has been characterized as the most salinity-tolerant species among emergent non-halophyte macrophytes, with a salinity tolerance limit of 12 g L^−1^ [249]. The recent comprehensive review summarized information on biology, ecology, invasion-related problems, as well as ecosystem services provided by *Typha* species [250], and readers are encouraged to refer to this source for detailed information. Therefore, only limited information on the halophytic nature of the species of this genus and its relationship to clonality will be presented here.

The rhizome expansion rate for both *Typha* species is extremely high, on average 26.5 and 31.0 cm per year for *T. angustifolia* and *T. latifolia*, respectively [3]; however, the expansion rate as high as 76 cm during single summer has also been observed [250]. It is suggested that *T. latifolia* plants allocate a relatively larger proportion of resources towards vegetative expansion by rhizomes, but *T. angustifolia* plants invest more in shoot growth [251,252]. However, rhizome longevity is higher in *T. angustifolia* [251].

When cultivated under alternating regimes of saline vs. freshwater pulse treatments, *T. latifolia* plants showed higher growth in comparison to permanently salt-treated plants [233]. It is evident that the presence of a large biomass of rhizomes with accumulated carbohydrate reserves acts as a main mechanism allowing the regrowth of *Typha* individuals after episodes of high salinity. *Typha* plants did not tolerate salinity during the establishment of seeds, as both seed germination and the growth of seedlings were extremely sensitive [253]. Established plants with rhizomes showed significantly higher salinity tolerance, but significant growth reduction was evident already at 5 g L^−1^; however, growth was completely inhibited at 25 g L^−1^ salinity followed by leaf decay at higher salinities. However, rhizomes appeared to be the most tolerant part of the plant, as the development of new shoots occurred after salinity-induced leaf death.

Another species of the genus, *Typha domingensis*, native to South America and characteristic of relatively warm climates, has been also included in the eHALOPH database. *T. domingensis* plants have been frequently used in different types of constructed wetlands for phytoremediation purposes and can adapt to conditions of high salinity (8 g L^−1^) and high pH (10) [254]. The plants adapted to such conditions for three years showed signs of stress when exposed to low salinity (0.2 g L^−1^) and lower pH (7), but plants adapted to such conditions showed signs of stress under high salinity and pH conditions. Interestingly, salinity-adapted plants accumulated a lower amount of Na^+^ in leaves in comparison to non-adapted plants when cultivated at high salinity. *T. domingensis* accumulated 24 g Na^+^ kg^−1^ in shoots in non-saline soil, and this value increased to 41 g kg^−1^ at 300 mM NaCl, but the concentration of K^+^ gradually decreased with an increase in salinity from 27 to 21 g kg^−1^ [255]. A high degree of salt-induced accumulation of non-ionic osmolytes seems to be associated with good salinity tolerance of *T. domingensis* ecotypes [256].

### 4.20. Viccia cracca

**Figure 20:**
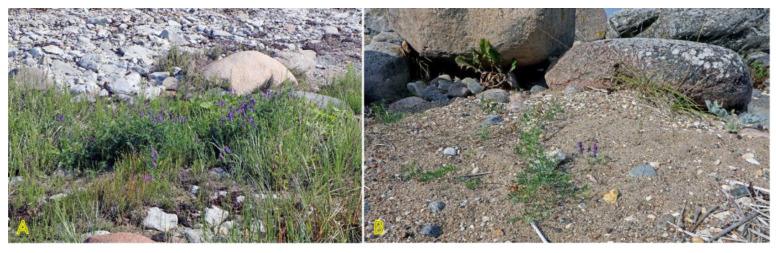
*Vicia cracca* in a coastal depression with perennial vegetation on a shingle beach on the island of Saaremaa, Estonia (**A**) and on a sandy beach on the island of Kihnu, Estonia (**B**).

A rhizomatous climbing legume species, *Vicia cracca*, has been relatively commonly found on stable gravel beaches as well as in coastal depressions with well-developed perennial vegetation on coasts of the Baltic Sea (Figure 20). In general, the species is typically found as a component of meadows, margins of forests, banks of rivers, and roadsides [257]. In Sweden, the species is characterized as moderately salinity tolerant [27]. No information is available on experimentally assessed salinity tolerance or aspects of clonal growth in *V. cracca*. However, Na^+^ accumulation potential in leaves of *V. cracca* from salt-affected habitats was extremely low, and plants evidently control EC levels in leaf tissues by regulating K^+^ concentration [29].

## 5. Halophyte Species with Bud-Bearing Roots

### 5.1. General Aspects

Among potential clonal halophytes, only three species were with bud-bearing roots as a dominant CGO: *Artemisia maritima* (Compositae), *Lepidium latifolium* (Brassicaceae), and *Linaria vulgaris* (Plantaginaceae). *Eryngium maritimum* (Apiaceae) was also included in this group as a special case of root-associated clonality. In addition, *Plantago maritima* (Plantaginaceae), initially not included in the list, was mentioned with respect to possible clonality. Information on *Ambrosia psilostachya* (Compositae), a species native to North America, was included due to invasiveness in coastal habitats in Europe.

An important feature of root-sprouting species is the ability to produce shoot-bearing buds not only at nodes but anywhere on the root system [3]; which, at least in theory, enables a more plastic response to environmental changes. Root-sprouting species make up about 10% of the Central European flora [258]. However, due to methodological problems in the identification of this type of clonal growth in natural conditions, it seems that the list of these species might be incomplete. Similarly, the list of characteristic clonal growth subtypes of species with bud-bearing roots might not be exhaustive. Sometimes, it seems that the presence of horizontally oriented roots are been mistaken for rhizomes, as in the case of *Ambrosia psilostachya* and *Lepidium latifolium*.

One of the most important differences between plants with shoot-based clonal growth organs (stolons and rhizomes) and those that use roots is related to the possibility of sensing the chemical composition of the soil in the second case [259]. While adventitious roots can be established on stolons and rhizomes only after their elongation and formation of ramets, elongating bud-bearing roots, in principle, can sense soil continuously and can appropriately form buds for shoot ramet placement and growth.

### 5.2. Ambrosia psiolstachya

In its native range, *Ambrosia psilostachya* is characteristic of relatively open habitats on sandy soils, such as prairie grasslands [260]. The species has become naturalized or invasive in Europe and Asia [261]. In France, *A. psilostachya* plants mostly colonize ruderal (habitats (61%), followed by agricultural lands (14%) and degraded sandy grasslands and dunes (12%)) [262]. In contrast to other species of the genus, *A. psilostachya* plants are perennial, possessing bud-bearing branching roots [263]. Some studies, however, incorrectly indicate the species as “rhizomatous” [264,265]. The potential for clonal expansion is high, as plants originally having single shoots can expand up to 2 m^2^ two years after the establishment [266].

In Sweden, *A. psilostachya* is characterized as a moderately salinity-tolerant species, relatively often appearing in seashore habitats [27]. However, in natural habitats of saline flats and basins *A. psilostachya* can occupy soil patches of extreme salinity if these are located adjacent to patches with less intense salinity [265]. It was identified in controlled conditions that plants were able to sustain the growth of ramets located in high salinity conditions due to resource allocation from ramets located in non-saline patches [264]. Interestingly, clonal growth was extremely restricted in non-saline soil (3% of ramets located at 20 cm or farther from parent ramets) in contrast to saline soil (28% of ramets located at 20 cm or farther from parent ramets).

### 5.3. Artemisia maritima

**Figure 21:**
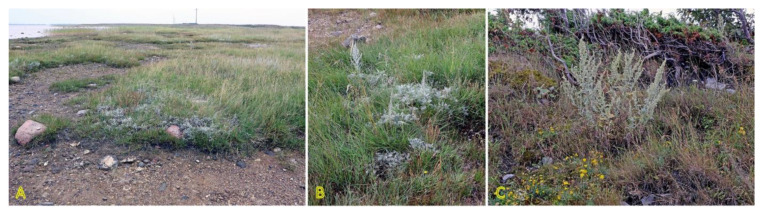
*Artemisia maritima* plants on a gravel beach on the island of Saaremaa, Estonia (**A**,**B**). *Artemisia absinthium* on a beach on the island of Saaremaa, Estonia (**C**).

Several species of the genus *Artemisia* are frequently found in salt-affected coastal habitats (Figure 21). Among them, only some have clonal growth potential [3]. *Artemisia maritima* is a species with a specific location in salt marsh vegetation in Europe [267]. *A. maritima* has bud-bearing roots with a relatively high potential for clonal expansion [3], and it is listed in the eHALOPH database. In Sweden, the species is characterized as “competitive only under moderate–high salinity” (indicator value 4 out of 5) [27]. Similar to the other species of the genus, *A. maritima* has very high pharmacological potential [268].

However, available information on the ecology and ecophysiology of *A. maritima* is extremely limited. The species has a prominent competitive ability under high nitrogen availability and becomes dominant at later stages of salt marsh succession [269]. In another study, the effects of experimental fertilization in salt marsh sites with different successional stages were assessed, and it was found that *A. maritima* positively responded to nutrient addition [270]. It is interesting to note that in natural conditions of salt-affected habitat, *A. maritima* showed a high potential for Na^+^ accumulation in leaves, reaching 43.3 g kg^−1^ DM [29].

### 5.4. Eryngium maritimum

*Eryngium maritimum* is a coastal-specific plant species with a characteristic xerophytic morphology. The species is included in the eHALOPH database and is characterized as “favoured by moderate salinity, but not restricted to such habitats” in Sweden [27]. While the presence of bud-bearing roots is indicated for *E. maritimum*, it is not characterized as clonal [3]. However, several researchers have indicated that the species possesses characteristics of clonal growth [271,272,273]. However, there does not seem to be sufficient experimental evidence at present to classify this species among the clonal halophytes. A comprehensive review of the biology and ecology of *E. maritimum* has been published [274].

### 5.5. Lepidium latifolium

*Lepidium latifolium* is a long-lived perennial with a high potential for both generative and vegetative reproduction. Within only two seasons, a single established individual can occupy an area several meters in diameter [275]. This probably is one of the reasons why the species has become invasive in the United States spreading through wetland and riparian habitats [276]. A study conducted under field conditions in sites with different moisture regimes and soil salinities (drier freshwater site, brackish marsh with nearly saturated soil, highly saline wet soil) has revealed that neither plant height nor inflorescence number was affected by salinity or moisture levels, but seed production and viability were negatively affected by the increase of both factors [277]. Plant density was reduced only in the high-salinity site. Clonal characteristics were not assessed in this study, but it was hypothesized that both salinity and soil waterlogging-related anoxia could have limited vegetative expansion.

**Figure 22:**
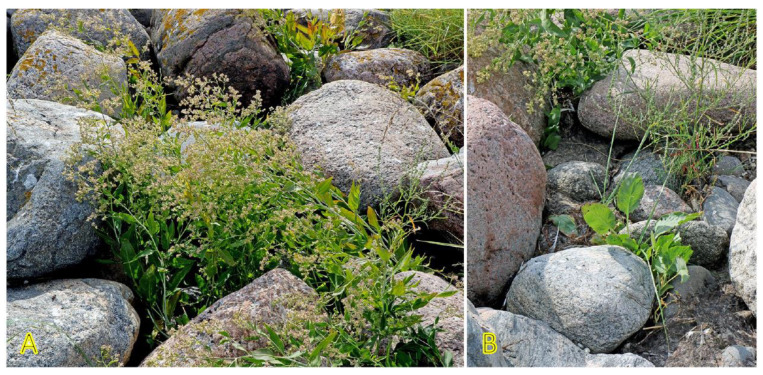
*Lepidium latifolium* plants on boulder beach on the island of Kihnu, Estonia. (**A**), established individuals; (**B**), young individual.

In contrast to being invasive species in the United States, in other regions, *L. latifolium* is considered to have potential as functional food or nutraceutical [278] or as a donor species for stress–tolerance engineering [279]. In the Baltic Sea region, plants can be found on different types of beaches in relatively stable substrate conditions (Figure 22). The species is included in the eHALOPH database and is characterized as “competitive only under moderate–high salinity” (indicator value 4 out of 5) [27].

In conditions of sand hydroponics, growth of *L. latifolium* plants was not negatively affected by irrigation with 48 mM NaCl (electrical conductivity of solution reaching 6.2 dS^−1^), but leaf area tended to decrease [280]. At this salinity level, Na^+^ was predominantly accumulating in leaves (44.5 g kg^−1^ DM) and stems (10.4 g kg^−1^ DM), and at a relatively low concentration in roots (4.2 g kg^−1^). Interestingly, it was noted that moderate salinity induced root bud development with the formation of shoots at the distal ends of the roots [280]. However, the experimental conditions did not allow the evaluation of the effect quantitatively.

In another study, involving cultivating *L. latifolium* plants in conditions of aerated hydroponics, shoot growth was inhibited at 300 and 400 mM NaCl for one week by 43 and 45%, respectively [281]. Na^+^ concentration in leaves reached 130 g kg^−1^ DM at 400 mM NaCl, but K^+^ concentration decreased to <50 g kg^−1^ DM, from 165 g kg^−1^ in control plants. Within three weeks, Na^+^ concentration in young and old leaves reached 224 and 301 g kg^−1^ DM, respectively, which was associated with an increase in leaf thickness and their water content. Therefore, the species has been designated as a typical accumulating halophyte.

### 5.6. Linaria vulgaris

**Figure 23:**
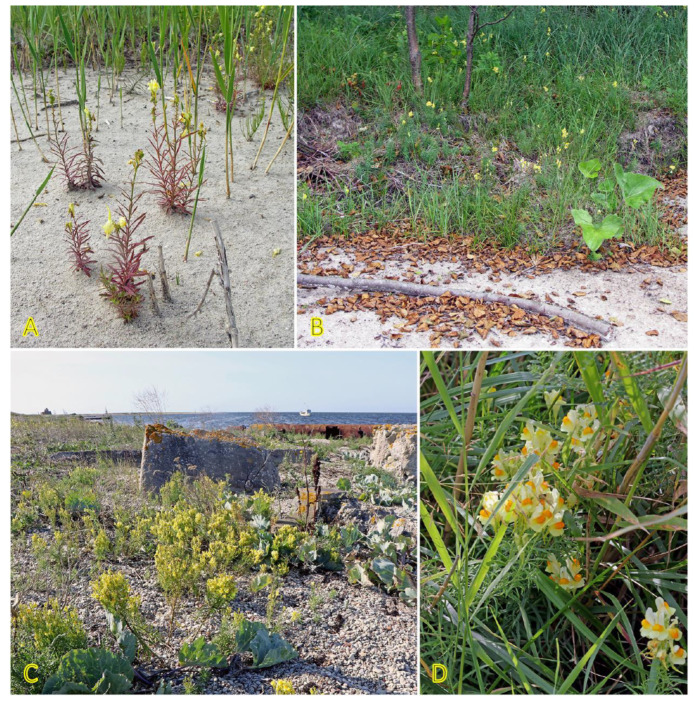
*Linaria vulgaris* plants on a sandy beach in Mērsrags, Latvia (**A**), on a forest edge near a wet sandy beach in Melnsils, Latvia (**B**), on a shingle beach on the island of Saaremaa, Estonia (**C**), on a wet coastal meadow on the island of Kihnu, Estonia (**D**).

*Linaria vulgaris* is a species with relatively abundant representation in coastal habitats. It can be found in small groups on the edges of wet sandy beaches, relatively large groups on gravel and pebble beaches, and even in coastal meadows (Figure 23), and it has been characterized as moderately salinity tolerant (indicator value 2 out of 5) [27]. The main clonal growth organs of *L. vulgaris* are bud-bearing roots capable of lateral expansion [3], and plants possessing a relatively high level of clonal integration [282].

The species has an extremely high potential for propagation by detached clonal root fragments. Thus, 10 cm long root explants bearing 10 cm high shoots were able to produce a genet 2 m in diameter during only one vegetation season [283]. Even short segments of roots (about 1 cm) were able to produce shoots due to the abundant presence of dormant vegetative buds along roots, becoming growth-competent due to wounding [284]. Radial expansion of *L. vulgaris* by lateral roots can be as fast as 1.2 m per year [285].

The species has been used to check whether root-sprouting clonal plants directly react to environmental heterogeneity [259]. It was found that more biomass was produced in heterogeneous environments in comparison to that in homogeneous environments, and more root biomass was found in nutrient-rich substrate patches. However, the number of ramets did not change with respect to nutrient availability in heterogeneous environments. So far, no information has become available on the experimentally assessed salinity tolerance of *L. vulgaris*. It would be especially interesting to compare salinity responses of different accessions including habitats with different substrate salinity levels, and to find out if clonal growth and vegetative propagation characteristics are affected by increasing salinity.

### 5.7. Plantago maritima

**Figure 24:**
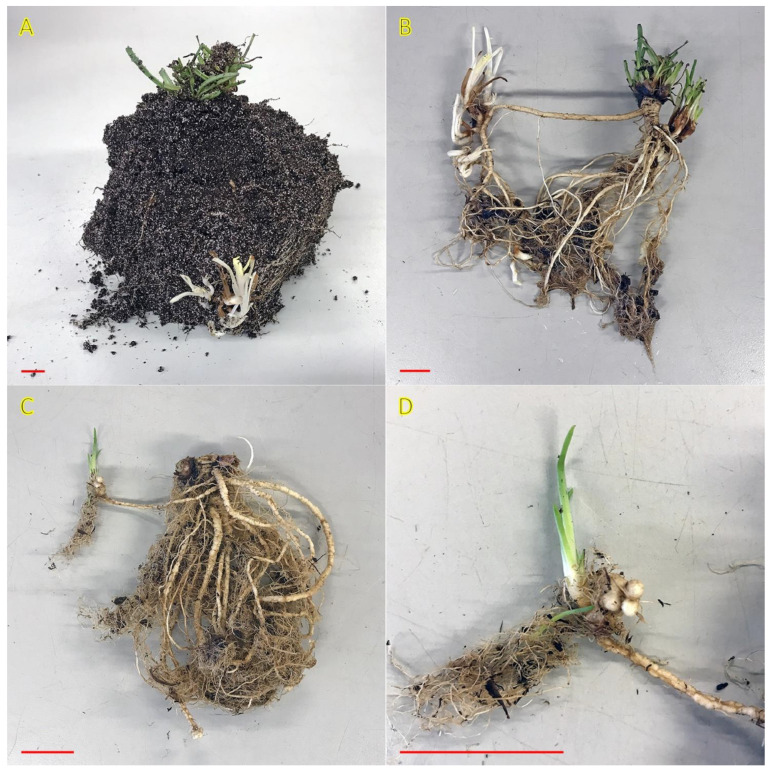
Presence of shoots and tuber-like structures on roots of *Plantago maritima* plants cultivated in controlled conditions in presence of 5 g L^−1^ Na^+^ in a form of chloride. (**A**), defoliated individual taken out from a container together with a substrate with an etiolated shoot at the lower corner of the container. (**B**), the same plant as in (**A**), with the substrate removed. (**C**), defoliated plant with the substrate removed showing shoot emerged aboveground. (**D**), the same shoot as in (**C**) with tuber-like structures. Bar indicates 10 mm.

While being a typical halophyte species, *Plantago maritima* has not been characterized as having any organ of clonal growth [3]. However, during a study of the comparative salinity tolerance of *P. maritima* to NaCl and KCl [286], it was found that several *P. maritima* plants developed shoots on vertically or horizontally oriented roots situated in the soil at the depth of the container (Figure 24). In addition, the presence of tuber-like structures was evident (Figure 24D). It would be necessary to investigate whether such a phenomenon is also observed under natural growth conditions and whether it could be a manifestation of salinity-induced clonality. It is important to note that the two other species of the genus, *Plantago lanceolata* and *Plantago media*, represent typical root-sprouting species [258].

## 6. Conclusions and Future Perspectives

### 6.1. Development of Suitable Model Systems for Functional Studies

Mostly ecological aspects of clonal plant biology have been assessed so far, including even effects of clonality at the level of ecosystem functions [20]. Functional aspects of clonality in the salinity tolerance of halophytes have been rarely assessed. One of the problems in this respect is a lack of suitable model systems. One of the experimental problems related to studies of clonal halophytes is related to the fact that many dicotyledonous species develop clonal characteristics only in the third year after establishment from seeds. Therefore, very often, plant material collected in natural conditions has been used for propagation of experimental material, but the environmental history of parent plant material could have a very large effect on salinity responses during the following experiment [150].

As a result of the present analysis, some promising model systems for studying clonal aspects of salinity adaptations have emerged. *Calystegia sepium* is a rather unique model among clonal halophytes, as it is a climbing species possessing two mutually complementary CGO systems, based on long-lived hypogeogenous rhizomes acting as a lateral expansion system, and relatively short-lived plagiotropic stems, which penetrate soil in the autumn and form below ground overwintering rhizomes. The climbing strategy of herbaceous plants had been discussed recently from the point of possible structural parasitism, allowing them to invest relatively less biomass in stems, but the possibility of the existence of such a strategy was rejected [287]. Nevertheless, further studies with climbing clonal halophytic species would give a unique opportunity to differentiate salinity effects at multiple levels of functional organization. So far, it was shown that an accession of *C. sepium* from salt-affected coastal habitats can allocate relatively more biomass in rhizome growth with increasing salinity [150].

Other potentially interesting model species could include but are not limited to *Honckenya peploides* and *Leymus arenarius* (changes of clonal architecture as a result of an interplay between salt spray and sand accretion), *Linaria vulgaris* (clonal growth characteristics and salinity tolerance of ecotypes from habitats with different salinity level), *Potentilla anserina* (coastal-specific ecotypes with different clonal growth characteristics), *Trifolium fragiferum* (role of salt-tolerant, symbiotic nitrogen-fixing rhizobacteria in clonal expansion) [288], *Tussilago farfara* (effect of salinity on clonal propagation ability of accessions from habitats with different salinity levels).

### 6.2. Ecological Role of Clonality in Coastal Habitats

Functional diversity vs. habitat diversity. Some potential clonal halophyte species show a surprisingly wide diversity range with respect to habitats in which they are located natively (*Agrostis stolonifera, Lysimachia maritima, Potentilla anserina, Trifolium fragiferum, Tripolium pannonicum*). In the case of species with rapid clonal expansion capability, such as the ones having stolons or epigoegenous rhizomes, such a phenomenon could be associated with greater phenotypic plasticity, manifested both at the morphological (as changes in clonal growth organ architecture) and biochemical levels (as spatial changes in resource storage and utilization), initiated by differences in environmental conditions.

Besides salinity tolerance, some species possess a high degree of adaptation to other prevalent environmental constraints, such as sand accretion for beach and dune species (*Honckenya peploides, Leymus arenarius*), flooding tolerance for wetland species (*Agrostis stolonifera*, *Bolboschoenus maritimus, Schoenoplectus tabarneamontani, Tripolium pannonicum, Typha* spp.), or grazing tolerance for shore meadow species (*Juncus gerardii*). Thus, at least some clonality-related characteristics of these species could represent integrative adaptive traits allowing buffering of the impact of changes in complex conditions while maintaining functional resilience.

Detailed analysis of habitat types where various clonal halophyte species are found was not the purpose of this review, but it can be seen from the provided examples that many of these species had a very wide ecological niche possibly associated with clonal growth characteristics. Similarly, information on phytosociological aspects was not included mostly due to the lack of comprehensive studies. However, several examples from studies aimed at understanding halophytic plant communities in the Baltic Sea region as well as in inland salt marshes can be found [28,30,177]. On the other hand, scientific interest in plant communities dominated by clonal plants and their ecological importance has increased recently [3,6,8,9,11,12]. There is no doubt that more ecophysiological studies need to be performed within the framework of halophytic and salt-tolerant species with the clonal type of growth. Most importantly, morphophysiological differences related to habitat type and plant communities need to be analyzed. Although the focus of this review was on coastal salt-affected habitats, undoubtedly, the information on clonal halophyte species from different inland saline habitats can be further incorporated into the analysis.

### 6.3. How Salinity Affects Clonal Growth Characteristics?

Some of the plants analyzed in the present review have shown a significant effect of salinity on their clonal growth characteristics. This evidence has been obtained both in field surveys and under controlled conditions, but its fragmentary nature does not allow general conclusions to be drawn. There is no doubt that the strategy of placement of ramets is affected by heterogeneous salinity in field conditions, as shown for *Ambrosia psilostachya* [265], *Hydrocotyle bonariensis* [102], *Spartina patens* and *Distichlis spicata* [170], *Leymus chinensis* [201]. It seems that resource allocation between clonal vs. photosynthesizing structures or storage structures is affected by salinity, as shown both in field studies and experiments in controlled conditions. Thus, *Phragmites australis* allocate more biomass to clonal growth in higher salinity [226]. Also, the growth of *Carpobrotus edulis* was significantly stimulated, including stimulation of the formation of new shoots, at moderate salinity [53].

### 6.4. Does Salinity-Induced Clonality Exist?

The problem with induced clonality itself has not been assessed in detail in the recent literature. Usually, the term has been used in an ecological context in order to describe the effect of subtly changing factors (disturbances) on clonal growth frequency in a certain habitat [289]. The phenomenon of “enforced clonality” has been analyzed experimentally and its adaptive importance in disturbed habitats was shown [290]. It is possible that salinity-induced clonality is characteristic of the root-sprouting species *Lepidium latifolium* [281]. In addition, fragmentation of CGO by natural or anthropogenic factors can lead to significant changes in clonal plant distribution. This mechanism can be an important clonal characteristic of coastal plants with fragmenting and dispersible rhizomes, distributed by water, as shown for several typical wetland species.

However, there is an assumption that non-clonal plant species can develop some type of CGO in special situations. It has been suggested that the seasonal appearance of new shoots of *Eryngium maritimum* depends on growth initiation in dormant vegetative buds on roots at different depths relative to the soil level, which causes the shoots to “move” relative to where they were situated in the previous season [273]. However, there is no reason to believe that this process is associated with substrate salinity.

### 6.5. How Modularity and Clonality Affect Ion Accumulation in Salt-Avoiding and Salt-Accumulating Halophyte Species?

The strategy of ion homeostasis is an important component in salinity tolerance mechanisms; especially, in halophytic species. Usually, halophyte species either avoid salt accumulation in aboveground organs, by storing ions in roots or releasing them outside the leaves, or use a strategy of ion accumulation by sequestration in specific tissues and cell compartments. Even for non-clonal species, patterns of ion accumulation change between modules of different ages, as well as in different organs. The majority of analyzed potential clonal halophyte species had low salt accumulation potential in aboveground parts, indicating them as typical excluders. It seems that even salt-accumulating species exclude Na^+^ from actively growing clonal structures, such as stolons for *Trifolium fragiferum* [85] and rhizomes for *Calystegia sepium* and *Calystegia soldanella* [150]. Osmotic equilibration is another problem; especially, in the case of salt-accumulating species with pronounced gradients of ion accumulation. One can expect that if Na^+^ is used as osmotica in leaves, other types of osmolytes need to be stored in non-salt-accumulating plant parts.

### 6.6. How Epigenetic Control in Clonal Plants Affects Their Responses to Salinity?

As already analyzed, some studies use clonally propagated plant material from natural accessions or even transplants from natural habitats. Conclusions based on the results from these types of studies need to be addressed with extreme caution. For example, if vegetatively propagated plant material from different zones of a habitat along a gradient of a certain environmental factor was used, and existence of ecotypic differences has been claimed, it is highly likely that some type of “stress memory” has been encountered.

Transgenerational memory through epigenetic regulation in clonal plant species is highly possible [291]. For example, salinity can increase epigenetic diversity in the population of invasive clonal species *Alternanthera philoxeriodes*, contributing to the evolutionary potential of the species [292]. Detailed analysis of these aspects is out of the scope of the present review, but there are several relatively recent discussions with respect to the role of epigenetic memory in clonal plants [291,293] as well as in plant responses to abiotic factors, including salinity [294,295,296,297].

## Figures and Tables

**Table 1:** The list of potential clonal halophyte plant species of the Baltic Sea region.

Family	Species (Synonym)	Presence in eHALOPH ^1^	Clonal Growth Organ ^1^	Persistence (Years) ^1^	Offspring Number (per Year) ^1^	Spread (cm) ^1^	Salinity ^2^	Sandy/Stony/Rocky Sea Shores ^2^	Sea Shore Meadows ^2^	ECe (dS m^−1^) ^3^
Monocotyledons										
Cyperaceae	*Blysmus rufus* (Huds.) Link	+	Rhizome H Rhizome E	4.0	1.0	6.8	4	0	10	na
Cyperaceae	*Bolboschoenus maritimus* (L.) Palla (*Scirpus maritimus* L.)	+	Rhizome H Stem tubers	3.4	6.4	32.1	3	0	7	63.7
Cyperaceae	*Eleocharis parvula* (Roem. & Schult.) Link ex Bluff, Nees & Schauer	+	Rhizome H Rhizome R Stem tubers	1.0	2.7	8.8	4	0	10	na
Cyperaceae	*Schoenoplectus tabernaemontani* (C.C.Gmel.) Palla (*Scirpus tabernaemontani* Gmelin) (*Scirpus validus* Vahl)	+	Rhizome H	4.0	1.0	40.0	4	0	7	15.6
Iridaceae	*Iris pseudacorus* L.	–	Rhizome E	4.0	2.7	8.8	1	0	0	na
Juncaceae	*Juncus balticus* Willd.	+	Rhizome H	4.0	4.3	13.0	4	7	0	na
Juncaceae	*Juncus bulbosus* L.	–	Rhizome E Rhizome H Stolons	3.0	3.5	14.0	1	0	0	na
Juncaceae	*Juncus compressus* Jacq.	–	Rhizome H	4.0	2.7	13.0	3	1	3	59.3
Juncaceae	*Juncus gerardii* Loisel.	+	Rhizome H	4.0	4.3	13.0	4	0	7	53.9
Juncaginaceae	*Triglochin maritima* L.	+	Rhizome E	4.0	1.0	0.5	4	3	7	84.2
Juncaginaceae	*Triglochin palustris* L.	+	Rhizome E Rhizome H Bulbs	1.5	4.3	6.8	2	0	0	12.9
Poaceae	*Agrostis stolonifera* L.	+	Stolons Rhizome E	1.8	5.0	13.0	3	0	2	118.0
Poaceae	*Elymus repens* (L.) Gould (*Agropyron repens* P.Beauv.) (*Elytrigia repens* (L.) Nevski)	+	Rhizome H Rhizome E	4.0	6.4	25.5	2	3	1	45.5
Poaceae	*Festuca rubra* L.	–	Rhizome H Rhizome E	4.0	3.8	6.8	2	1	1	25.7
Poaceae	*Leymus arenarius* (L.) Hochst.	+	Rhizome H Rhizome E	4.0	4.8	9.9	2	7	0	na
Poaceae	*Phragmites australis* (Cav.) Trin. ex Steud.	+	Rhizome H Stolons Rhizome E	4.0	3.1	30.9	2	0	3	118.0
Poaceae	*Puccinellia maritima* (Huds.) Parl.	–	Stolons Rhizome E	2.8	5.8	20.0	5	0	10	na
Typhaceae	*Typha angustifolia* L.	+	Rhizome H	4.0	4.8	26.5	2	0	0	na
Typhaceae	*Typha latifolia* L.	+	Rhizome H	3.5	5.0	31.0	2	0	0	7.50
Dicotyledons										
Apiaceae	*Eryngium maritimum* L.	+	Bud bearing roots	na	na	na	3	10	0	na
Araliaceae	*Hydrocotyle vulgaris* L.	–	Rhizome E Stolons	1.6	6.0	18.4	1	0	0	na
Brassicaceae	*Lepidium latifolium* L.	+	Bud bearing roots Rhizome H	4.0	1.0	11.5	4	9	0	na
Caryophyllaceae	*Honckenya peploides* (L.) Ehrh.	+	Rhizome H	4.0	4.3	13.0	4	10	0	na
Compositae	*Achillea millefolium* L.	–	Rhizome H Rhizome E	4.0	4.8	14.3	2	0	0	26.3
Compositae	*Artemisia maritima* L.	+	Bud bearing roots	4.0	4.3	13.0	4	0	10	27.5
Compositae	*Petasites spurius* (Retz.) Rchb.	–	Rhizome H Rhizome E	3.0	1.0	26.5	2	7	0	na
Compositae	*Tripolium pannonicum* (Jacq.) Dobrocz. (*Aster tripolium* L.)	+	Rhizome E Rhizome H	4.0	3.5	0.5	4	0	10	118.0
Compositae	*Tussilago farfara* L.	–	Rhizome H	2.7	5.1	29.9	2	0	0	na
Convolvulaceae	*Calystegia sepium* (L.) R. Br.	+	Stolons Rhizome H	1.0	6.0	34.6	2	1	1	20.1
Convolvulaceae	*Calystegia soldanella* (L.) Roem. & Schult.	+	Rhizome H	4.0	na	13.0	na	na	na	na
Leguminosae	*Lathyrus japonicus* Willd. (*Lathyrus maritimus* (L.) Bigelow)	+	Rhizome H	4.0	5.0	29.2	4	10	0	na
Leguminosae	*Trifolium fragiferum* L.	+	Stolons	1.7	1.8	16.9	3	0	7	11.5
Leguminosae	*Trifolium repens* L.	–	Stolons Bud bearing roots	1.9	3.9	26.5	2	0	1	10.8
Leguminosae	*Vicia cracca* L.	–	Rhizome H Bud bearing roots	4.0	6.0	22.0	2	1	0	25.7
Plantaginaceae	*Linaria vulgaris* Mill.	–	Bud bearing roots Rhizome H	3.8	2.4	21.5	2	2	0	na
Primulaceae	*Lysimachia maritima* (L.) Galasso, Banfi & Soldano (*Glaux maritima* L.)	+	Rhizome H Stem tubers	2.0	4.8	13.0	4	3	7	118.0
Rosaceae	*Filipendula ulmaria* (L.) Maxim.	–	Rhizome H Rhizome E	3.7	2.7	10.9	2	2	0	na
Rosaceae	*Potentilla anserina* L. (*Argentina vulgaris* Lam.) (*Argentina anserina* (L.) Rydb.)	–	Stolons Rhizome E Bud bearing roots	2.4	3.6	23.9	3	1	2	22.2
Rubiaceae	*Galium verum* L.	–	Rhizome H Stolons	3.8	1.6	13.0	2	0	0	na

^1^ Data from Herben, Klimešova (2020) [3]. ^2^ Data from Tyler et al. (2021) [27]. ^3^ Maximum electrical conductivity of the soil-saturated extract in inland salt marshes (Piernik 2012) [30]. na, not available.

## Data Availability

All data are taken from published sources. All photographs are taken by the author.
